# Targeted monitoring informed by mapping the ongoing spread of tick-borne encephalitis virus, the Netherlands

**DOI:** 10.2807/1560-7917.ES.2026.31.20.2500767

**Published:** 2026-05-21

**Authors:** Sara R Wijburg, Dedeke Rockx-Brouwer, Megan K Herbert, Jolianne M Rijks, Paulina M Lesiczka, Miriam Maas, Andrea Gröne, Renate W Hakze-van der Honing, Maya Holding, Helen J Esser, Hein Sprong, Egil AJ Fischer

**Affiliations:** 1 National Institute for Public Health and the Environment (RIVM), Centre for Infectious Disease Control (CIb), Bilthoven, the Netherlands; 2 Dutch Wildlife Health Centre (DWHC), Pathology, Faculty of Veterinary Medicine, Utrecht University, Utrecht, the Netherlands; 3 The Netherlands Food and Consumer Product Safety Authority, Wageningen, the Netherlands; 4 Wageningen Bioveterinary Research, Wageningen University & Research, Lelystad, the Netherlands; 5 United Kingdom Health Security Agency (UKHSA) Diagnostics and Pathogen Characterisation Division, Porton Down, Salisbury, United Kingdom; 6 Wildlife Ecology and Conservation Group, Department of Environmental Sciences, Wageningen University & Research, Lelystad, the Netherlands; 7 Veterinary Epidemiology, Department of Population Health Sciences, Faculty of Veterinary Medicine, Utrecht University, Utrecht, the Netherlands

**Keywords:** Tick-borne encephalitis, host community, next generation matrix, reproduction number, *Ixodes*, public health, monitoring

## Abstract

**BACKGROUND:**

Tick-borne encephalitis virus (TBEV) was detected in the Netherlands in 2015. Current monitoring through serology in deer, tick collection and reported human cases, may underestimate areas of virus circulation. Including estimates of suitability of the habitat for TBEV can guide risk-based monitoring.

**AIM:**

We aimed to improve the accuracy of the TBEV distribution map by adding estimates of TBEV habitat suitability to guide targeted tick monitoring in newly identified risk regions.

**METHODS:**

Habitat suitability for TBEV was assessed by calculating the basic reproduction number (R_0_) for 1 km^2^ grid cells aggregating various data sources for tick hosts such as mice or voles, tick abundance and TBEV transmission. The importance of different tick hosts was determined in scenario analyses. The baseline scenario was validated against data on seropositive roe deer and TBEV-positive ticks and rodents.

**RESULTS:**

The suitability (R_0_ ≥ 1) was associated with tick habitat suitability and the abundance of competent host species. Variation in competent host densities had a greater effect on R_0_ estimates than incompetent hosts. Monitoring data corresponded well with model-predicted high-risk areas and confirmed further spread of TBEV.

**CONCLUSION:**

The TBEV suitability map is a useful tool for targeted sentinel surveillance by identifying new risk areas, informing local municipal health services of endemic areas for potential human exposure. Our results support targeted efforts for awareness, preparedness and outbreak response, even in regions where TBEV has not yet been detected. Integrating One Health monitoring with spatial modelling can strengthen preparedness in emerging TBEV regions.

**Key public health message**

**What did you want to address in this study and why?**

Tick-borne encephalitis is caused by the tick-borne encephalitis virus (TBEV) and can lead to neurological symptoms. Since its first detection in the Netherlands, TBEV has spread in an unpredictable manner. Relying solely on confirmed cases from humans and ticks likely underestimates the areas at risk of exposure. We aimed to identify areas suitable for sustained TBEV transmission.

**What have we learnt from this study?**

In areas where TBEV is spreading, mapping habitat suitability (i.e. areas more or less suitable for sustained transmission of the virus) proves to be more effective than relying on case data alone. The map identifies areas where sustained transmission is possible, even without reported cases. This approach enabled the detection of TBEV in previously unrecognised regions. It provides a more efficient basis for targeted monitoring.

**What are the implications of your findings for public health?**

Our findings justify increasing awareness and preparedness among local health services regarding the possible circulation of TBEV in regions previously considered unaffected. The TBEV suitability map offers a valuable new tool for the identification of priority areas in which to look TBEV in ticks and can thus contribute to identifying high-risk areas for human and animal exposure.

## Introduction

Tick-borne encephalitis (TBE) is a zoonosis and a growing public health concern because of its potentially severe clinical progression, lack of specific treatment options, increasing incidence in endemic regions and emergence in previously unaffected areas [1-4]. The causative agent, tick-borne encephalitis virus (TBEV), emerged relatively recently in the Netherlands. The first autochthonous human cases were identified in 2016, shortly after local circulation of the virus was confirmed in ticks (*Ixodes ricinus*) in 2015 from the Sallandse Heuvelrug region [4-6]. Subsequent infections were identified in humans and animals over the following years [7,8]. In early 2025, TBE was categorised as a notifiable disease in the Netherlands [9]. Integrated monitoring efforts – referred to as One Health monitoring (OHM) – have since intensified, combining data from humans, animals and environment [7,8,10]. Despite these efforts, uncertainties remain about where TBEV circulates and how it is sustained within the Dutch ecosystems. Addressing this knowledge gap will provide healthcare professionals and public health authorities with valuable information to support regional preparedness, improve early recognition and differential diagnosis of TBE in humans.

Identifying risk areas for TBEV is inherently challenging due to the complex ecology of the virus [11]. Tick-borne encephalitis virus is typically detected in hyper-localised environments (foci) where the principal tick vector (*I. ricinus*) and competent rodent hosts (i.e. species within the genera *Apodemus, Clethrionomys* and *Microtus*) coexist [11-15]. Medium to large-sized mammals, birds and lizards are considered incompetent hosts for TBEV transmission. However, some of these species, most prominently deer, play an essential role in the propagation and maintenance of tick populations at densities sufficient for virus circulation [16,17]. Moreover, roe deer are suitable for sentinel surveillance, as they develop antibodies against TBEV and have restricted stable home ranges [18,19]. The role of birds in the transmission of TBEV is not well understood, but they are considered to facilitate long-distance dispersal of the virus by transporting infected ticks [20].

Identification of new areas of emergence often relies on the discovery of human cases, a method limited by recall bias and underdiagnosis [21]. Alternative OHM approaches include detection of TBEV antibodies in wildlife and detection of viral RNA in ticks and/or rodents [7,8,10]. While monitoring of mammalian host species gives information about recent or current transmission of TBEV [7,8,10], this method has ethical and logistical challenges. Similarly, relying solely on tick collection to detect new TBEV foci is neither feasible nor cost-effective, due to the micro-focality of TBEV and low prevalence in ticks (often 0%, and < 5% in microfoci) [7,8]. Collecting ticks across broad landscapes can therefore be inefficient and may not yield meaningful results without prior knowledge of potential risk areas.

One Health monitoring can be strengthened by assessing the suitability of different areas (habitat) for TBEV transmission [22]. A useful metric for this is the basic reproduction number (R_0_), which estimates the potential for sustained transmission following pathogen introduction, and can be calculated even for areas where the pathogen is not yet detected [23]. However, due to the intricate ecology of TBEV with multiple hosts and transmission routes, calculating R_0_ is also complex [23]. This complexity can be addressed using the next generation matrix (NGM) approach, which categorises individual hosts according to their state at the time of infection (e.g. a tick infected as an egg via transovarial transmission). This allows for assessing differences in their epidemiological potential [23-25]. Although spatial mapping of R_0_ has been used for other vector-borne pathogens [26-28], it has yet to be applied to TBEV.

The National Institute for Public Health and the Environment (RIVM) provides a map of confirmed or probable TBEV microfoci and their corresponding Municipal Health Service (MHS, i.e. responsible for local public health across one or multiple municipalities) regions in the Netherlands, based on OHM data (https://www.rivm.nl/tekenencefalitis). However, the current map may not be detailed enough. Thus, we aimed to develop a spatially explicit modelling framework to calculate R_0_ for TBEV, incorporating host species abundance and spatial variation in tick-suitable habitat. The resulting area at risk map was validated using earlier OHM animal and environment data and newly collected animal data from 2024 to 2025. To further guide targeted tick monitoring for identifying TBEV microfoci, we aimed to detect the virus in MHS regions flagged as potential risk areas, using R_0_ values in combination with 2024–25 OHM data.

## Methods

**Figure 1:**
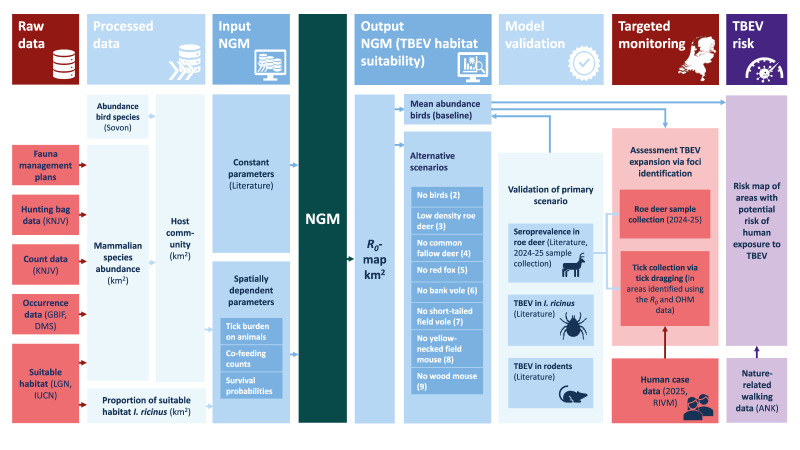
Workflow illustrating the integration of ecological data, host community structure and model simulations into a next generation matrix (NGM) framework to assess habitat suitability for tick-borne encephalitis virus, the Netherlands

ANK: Atlas of Natural Capital; DMS: Dutch Mammal Society; GBIF: Global Biodiversity Information Facility platform; *I. ricinus*: *Ixodes ricinus*; IUCN: International Union for Conservation of Nature and Natural Resources; KNJV: Royal Dutch Hunters' Association; LGN: National land-use database; R_0_: basic reproduction number; Sovon: Dutch Centre for Field Ornithology; TBEV: tick-borne encephalitis virus.

We modelled TBEV habitat suitability based on R_0_ at a 1 km^2^ resolution across the Netherlands. The workflow for quantifying and validating TBEV habitat suitability is shown in Figure 1. Detailed methods on the epidemiological model are described in Supplementary Text S3.

Relative importance of host species in TBEV transmission was evaluated across nine scenarios described in the figure.

Bird abundance data are available via Sovon (https://stats.sovon.nl/). Roe and fallow deer densities were derived from fauna management plans [55]. Mammal occurrence data are from GBIF (https://www.gbif.org/). Tick burden data and TBEV observations in deer, rodents, and ticks are from [6-18,30].

### Calculation of the basic reproduction number

Host community composition (processed data) was determined for each 1 km^2^ block using 18 species: competent hosts (mammals: yellow-necked field mouse (*Apodemus flavicollis*), wood mouse (*A*. *sylvaticus*), bank vole (*Clethrionomys glareolus*), short-tailed vole (*Microtus agrestis*)) and incompetent hosts (mammals: roe deer (*Capreolus capreolus*), fallow deer (*Dama dama*), red fox (*Vulpes vulpes*); birds: European stonechat (*Saxicola rubicola*), common blackbird (*Turdus merula*), tree pipit (*Anthus trivialis*), Eurasian skylark (*Alauda arvensis*), song thrush (*Turdus philomelos*), dunnock (*Prunella modularis*), Eurasian jay (*Garrulus glandarius*), common starling (*Sturnus vulgaris*), hawfinch (*Coccothraustes coccothraustes*), European robin (*Erithacus rubecula*) and common chaffinch (*Fringilla coelebs*)).

Spatial mean bird density was obtained from the Dutch Centre for Field Ornithology (Sovon; processed data). Mammalian density estimates were generated using a multi-source approach that combined hunting data (Royal Dutch Hunters' Association (KNJV); https://www.faunaregistratie.nl/default.aspx), systematic count data (KNJV), occurrence records (Global Biodiversity Information Facility platform (GBIF; https://www.gbif.org/) and Dutch Mammal Society (DMS; https://ipt.nlbif.nl/resource?r=zv-digitized-reports)), habitat suitability layers (International Union for Conservation of Nature and Natural Resources (IUCN; https://iucn.org/)) and/or fauna management plans (FMPs). The specific data-integration steps used to produce a density layer for each species included (raw data) are described in Supplementary Text S1 and summarised in Supplementary Table S2.

Data from the national land use database (LGN2021, 51 classes, 5 m resolution; raw data), were coded as suitable (1) or unsuitable (0) for ticks. The proportion of suitable habitat was then calculated for each grid cell and used to adjust tick-per-host estimates [29]. More details are described in Supplementary Text S2.

The R_0_ was calculated using the NGM according to previously described methods [23,30], where R_0_ was used as a measure of TBEV habitat suitability [23,24,31]. Host community composition and tick habitat suitability were combined with spatially independent parameters derived from literature (e.g. transovarial transmission probability [32], days of attachment tick [33] to inform the NGM (Input NGM). More details are presented in Supplementary Table S5. To understand the relative importance of different host species and groups for the TBEV transmission cycle, we modelled nine scenarios representing variations in host community composition: baseline with all host species and groups (1), as (1) but no birds (2), low density roe deer (3), no common fallow deer (4), no red fox (5), no bank vole (6), no short-tailed vole (7), no yellow-necked field mouse (8), and no wood mouse (9) (Output NGM).

### Model validation

The baseline scenario was validated using a randomisation test (10,000 repetitions) and OHM data. One Health monitoring data included information on TBEV detection in ticks, rodents and roe deer [6-8,10] and data on antibodies against TBEV in roe deer collected by the Dutch Wildlife Health Centre (DWHC) in 2024–25. Roe deer blood samples were submitted from hunted animals and fallen stock. Human case data were not included for validation purposes as no precise coordinates were available. The collected roe deer serum samples were tested for antibodies against TBEV using a commercial ELISA kit (EIA TBEV virus IgG, TestLine, Testline Clinical Diagnostics s.r.o. Brno, Czechia) according to the manufacturer’s instructions with two adaptations. We used another conjugate (Protein G*HRP conjugate, Thermo Fisher Scientific, Waltham, the United States (US)) and different cut-offs. The cut-off value for positive samples was determined as an optical density (OD) < 5 times the standard deviation (SD) of the OD values for the negative controls plus the average OD value of the negative controls. The cut-off value for borderline samples was OD 3 times the SD of the negative controls plus the average OD of the negative controls. Samples that tested positive in the ELISA were subsequently tested using a serum neutralisation test (SNT) [34]. The complete description of the randomisation procedure and the full OHM dataset are provided in Supplementary Text S4 and Table S8.

### Targeted monitoring

To evaluate potential TBEV spatial expansion in the Netherlands, TBEV foci identified in the roe deer survey of 2024–25 (were compared with previously reported foci [10].

For targeted virus monitoring, we selected 10 locations with R_0_ ≥ 1: nine with antibody-positive roe deer and one with GPS-tracked routes on human cases (Figure 1). We collected ticks from these 10 locations by dragging a 1 × 1 m cotton blanket over vegetation without using transects. The collected ticks were transported alive to RIVM and stored at −80°C before analysis.

The method to assess TBEV expansion based on roe deer samples and detection of TBEV in ticks were performed as previously described [6,10,35]. Further details are presented in Supplementary Text S5 and Figure S6.

To identify areas where TBEV habitat suitability overlaps with human recreational activity, we used nature-related walking data from the Netherlands (km/hectare/year) as a proxy [36,37]. Bivariate mapping was used for visualisation.

All analyses were conducted using ArcGIS Pro version 3.1.7 (https://www.arcgis.com/index.html) and R software version 4.4.2 (https://www.r-project.org).

## Results

### Suitability map

**Figure 2:**
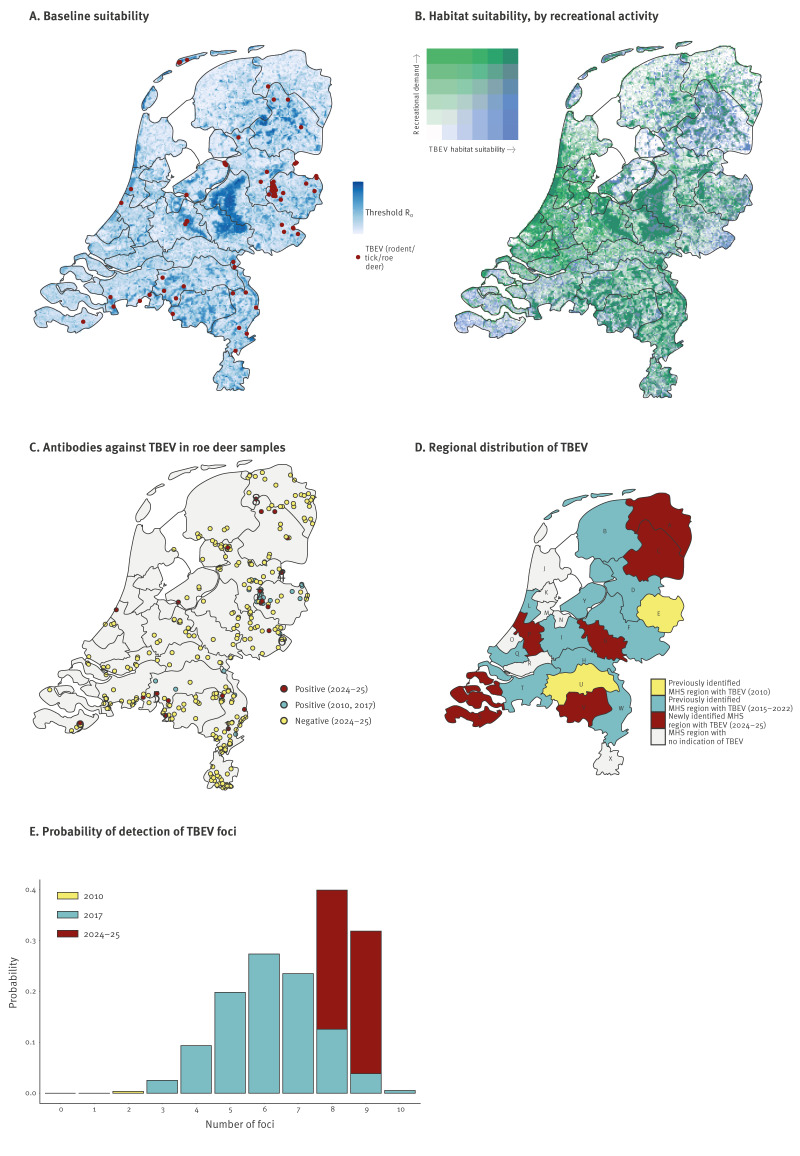
Spatial distribution, probability of detection and habitat suitability of tick-borne encephalitis virus, the Netherlands

MHS: Municipal Health Service; R_0_: basic reproduction number; TBEV: tick-borne encephalitis virus.

The baseline TBEV suitability map revealed considerable spatial heterogeneity in R_0_ values across the Netherlands, ranging from 0 to 2.27 and with 6,086 of 36,648 grid cells having an R_0_ ≥ 1 (Figure 2A). All 25 MHS regions included grid cells with a predicted R_0_ ≥ 1. Higher R_0_ values were observed in areas with greater suitability for the vector *I. ricinus* and higher abundances of competent hosts, particularly in the Veluwe area (52°5′N, 5°48′E) and National Park Utrechtse Heuvelrug (52.025°N, 5.437°E). The density of competent hosts and habitat suitability layers can be found in Supplementary Figures S3-S4. We further identified areas with a high TBEV habitat suitability and relatively high recreational activity (Figure 2B).

Borders in the maps (Panel A-D) represent those of the MHS regions.

Panel A. The R_0_ map of TBEV where R_0_ represents the average number of new infections. Red dots indicate detection of TBEV from ticks and rodents and seropositive samples from roe deer.

Panel B. Bivariate map displaying the overlap between recreational activity (standardised between 0 and 1) and TBEV habitat suitability. Nature-related recreation (walking) in the Netherlands (2018) was used as a proxy for recreational activity [37]. Green areas have a higher recreational activity and a lower TBEV habitat suitability. Blue areas have a higher TBEV habitat suitability and a lower recreational activity. Dark green areas have both a high recreational activity and a high TBEV habitat suitability.

Panel C. Antibody testing was done using a commercial ELISA test. Source: 2010–2017 data [6,10] and current study.

Panel D. Overview of TBEV status across MHS regions in the Netherlands. The Safety and Health Region Gelderland-Midden was identified based on human case data (2025), but no coordinates were available. The identification numbers represent the MHS regions listed in Table 1.

Panel E. Probability distribution of the potential number of TBEV foci.

### Monitoring

The DWHC received 322 roe deer serum samples between 2024 and 2025. Of the 312 samples that could be included in the analyses, 25 (8.0%) were seropositive for TBEV in ELISA, 13 (4.2%) were borderline and 274 (87.8%) were seronegative (Figure 2C). Based on these roe deer data results, along with human case data from 2025, we identified six new MHS regions with evidence of TBEV circulation. Thus, 18 of the 25 MHS regions have currently evidence of the presence of TBEV (Figure 2D). Under the baseline scenario, the spatial distribution of R_0_ was significantly associated with TBEV-positive OHM data (83/100 samples in grid cells with predicted R_0_ ≥ 1; randomisation test, p < 0.0001). In Supplementary Table S8, OHM data records and corresponding calculated R_0_ values are shown. The remaining observations that fell outside the predicted R_0_ ≥ 1 areas (17/100) were mostly data of seropositive roe deer (12/17).

Of the 25 seropositive roe deer samples, 17 were available for confirmatory testing with SNT. Fourteen of these samples tested positive with SNT; of the six newly identified TBEV-positive MHS regions, three had roe deer positive by both ELISA and SNT, one was identified from human case data, one had only sample available for SNT, and one could not be tested with SNT. Based on the TBEV-positive SNT locations, nine TBEV foci were identified (Figure 2A, Table 1). Based on these small numbers of samples, the probability of detecting TBEV foci was higher in 2024–25 compared with 2010 and 2017 (Figure 2E) [6,10]. All previously identified MHS regions were reconfirmed.

**Table 1:** Presence of antibodies against tick-borne encephalitis virus in serum samples of roe deer (*Capreolus capreolus*), by region, and the identification of foci in the Netherlands, 2024–25

ID^a^	MHS region	SNT 2010^b^			SNT 2017^b^		SNT 2024–25^c^			ID of foci identified 2024–25^c^
		Pos	Tested		Pos	Tested	Pos	Tested		
A	MHS Groningen^d^	0		27	0	32	1		1	8
B	MHS Fryslân	0		26	0	64	NA			NA
C	MHS Drenthe^d^	0		52	0	97	0		1	NA
D	MHS IJsselland	0		23	2	35	1		2	4
E	MHS Twente	5		17	7	52	5		5	7
F	MHS North and East Gelderland	0		41	2	67	1		1	6
G	Safety and Health Region Gelderland-Midden^d^	0		20	0	18	NA			NA
H	MHS South Gelderland	0		2	0	15	NA			NA
I	MHS Region Utrecht	0		5	0	13	NA			NA
J	MHS Northern Holland	NA			NA		NA			NA
K	MHS Zaanstreek-Waterland	NA			NA		NA			NA
L	MHS Kennemerland	NA			NA		NA			NA
M	MHS Amsterdam	NA			NA		NA			NA
N	MHS Gooi and Vechtstreek	NA			NA		NA			NA
O	MHS Haaglanden	NA			NA		NA			NA
P	MHS Central Holland^d^	NA			NA		0		0	NA
Q	MHS Rotterdam-Rijnmond	NA			0	5	NA			NA
R	South Holland South Joint Health Service	NA			0	2	NA			NA
S	MHS Zeeland^d^	NA			0	8	2		2	9
T	MHS West Brabant	0		10	2	30	1		2	3
U	MHS Heart of Brabant	1		17	1	33	1		1	5
V	MHS South-east Brabant^d^	0		24	0	38	1		1	2
W	MHS Northern Limburg	0		13	3	31	1		1	1
X	MHS South Limburg	0		2	0	15	NA			NA
Y	MHS Flevoland	0		18	0	37	0		0	NA
Total		6		297	17	592	14		17^e^	9

ID: identification number; MHS: municipality health service region; NA: not applicable; Pos.: positive; SNT: serum neutralisation test.

^a^ IDs correspond to numbering in Figure 2D.

^b^ Source [10].

^c^ Testing for antibodies was performed with a commercial ELISA. Samples positive in the ELISA were confirmed with serum neutralisation test. The IDs correspond to numbering in Figure 2C.

^d^ Newly identified MHS region based on antibody results of roe deer samples or human case data (2024–25).

^e^ Of the 312 samples analysed, 25 were seropositive in ELISA. Seventeen of the 25 samples were further analysed with serum neutralisation test.

We collected 4,787 questing ticks, including 401 adults (176 females and 225 males), 4,345 nymphs and 41 larvae. Ticks were analysed in altogether 252 pools based on life stage and sex: nymphs and larvae were pooled in groups of 25, males in groups of eight and females in groups of four. One (0.4%) pool tested positive for TBEV. This pool contained four female ticks and originated from a location in Zeeland, a newly identified MHS region, as presented in Supplementary Figure S6.

### Impact of host density variation on the basic reproduction number

**Figure 3:**
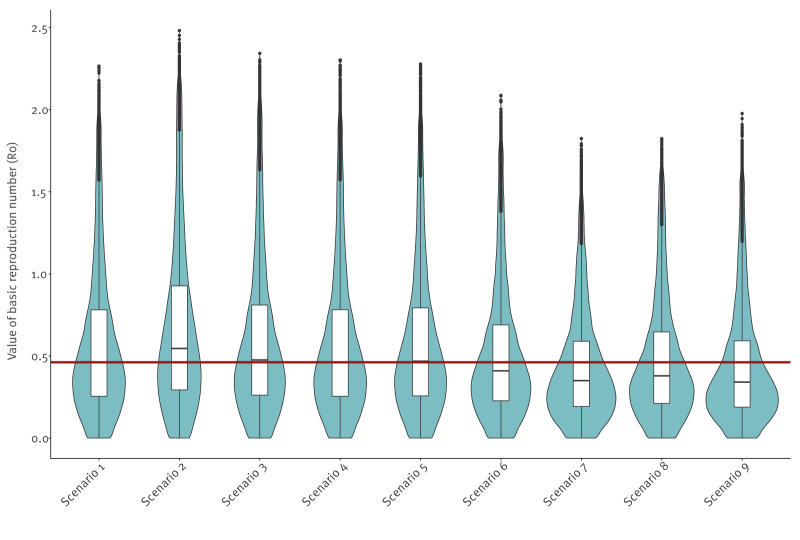
Distribution of the basic reproduction number of tick-borne encephalitis virus per scenario, the Netherlands

Birds exhibited the strongest inhibiting effect on R_0_: excluding them increased the overall median R_0_ in the Netherlands from 0.46 (IQR: 0.53) to 0.55 (IQR: 0.63), and the number of risk grid cells (R_0_ ≥ 1) rose 1.3-fold, from 6,086 to 8,087. In contrast, scenarios without competent rodent species (e.g. *Apodemus* spp., *Clethrionomys glareolus*, *Microtus agrestis*) showed an amplifying effect of rodent abundance, as the median R_0_ decreased to 0.34–0.41 and the number of risk grid cells declined 1.3–2-fold. Scenarios excluding larger mammals such as roe deer, common fallow deer and red foxes showed only minor changes in the value of R_0_ and in the number of grid cells with R_0_ ≥ 1 (Table 2, Figure 3).

**Table 2:** Relative importance of various host species within the tick-borne encephalitis virus transmission cycle, the Netherlands

Scenario ID	Scenario	Median R_0_	IQR	Percentage grid cells with R_0_ ≥ 1
1	Mean bird abundance (baseline scenario)	0.46	0.53	16.6%
2	No birds	0.55	0.63	22.1%
3	Low density of roe deer (*Capreolus capreolus*)	0.48	0.55	17.9%
4	No common fallow deer (*Dama dama*)	0.46	0.53	16.7%
5	No red fox (*Vulpes vulpes*)	0.47	0.54	17.0%
6	No bank vole (*Clethrionomys glareolus*)	0.41	0.46	12.8%
7	No short-tailed field vole (*Microtus agrestis*)	0.35	0.40	8.24%
8	No yellow-necked field mouse (*Apodemus flavicollis*)	0.38	0.44	11.2%
9	No wood mouse (*A. sylvaticus*)	0.34	0.40	9.48%

ID: identification number; IQR: interquartile range; R_0_: basic reproduction number.

The horizontal dark red line is used to show the median value of the baseline scenario (scenario 1).

## Discussion

The geographical range of TBEV continues to expand within the Netherlands, with evidence of emergence in ticks and other wild animals in previously unaffected regions as well as a newly notified human case in these regions. Because TBEV is still spreading, relying solely on OHM data may provide a limited representation of TBEV circulation, as such data are constrained by sampling effort and coverage. Human case data, for example, are inherently insensitive, representing only the “tip of the iceberg” of actual virus circulation, and cases may go undetected in areas where TBEV is assumed absent. Similarly, in regions with low deer density or limited collaboration with local hunters, serosurveillance may not be feasible. Thus, in countries or regions with few reported cases and/or where TBEV is still emerging, traditional surveillance alone could be insufficient. We therefore developed a comprehensive framework integrating multiple existing approaches to generate a risk map identifying areas potentially suitable for TBEV circulation, thereby guiding targeted monitoring efforts. This approach provides insights into the suitability of regions without confirmed circulation and is not restricted to TBEV and could thus be applied to other emerging pathogens (e.g. West Nile virus) (Box).

Box **Anticipated value of tick-borne encephalitis virus modelling approach to guide monitoring and intervention practices, the Netherlands**

**Functionality of the framework^a^**:

• Hazard: measure of national TBEV habitat suitability (R_0_) averaged over multiple years,

• Risk: TBEV habitat suitability combined with human recreation areas,

• Preparedness: overview of TBEV status of MHS regions,

• Spread: assessment of TBEV expansion,

• Extrapolation: could be applied in other countries where TBEV is emerging and to other pathogens.

**Epidemiological interpretation:**

• Wildlife and human exposure hazard to TBEV,

• Relative recreational exposure risk to TBEV.

**Public health actions that could be informed:**

• National:

o Suitability maps can guide a shift from case-based surveillance to a risk-based approach. The model identifies high-risk cells (R_0_ ≥ 1), therefore allowing sentinel surveillance and tick-dragging efforts to be concentrated beyond regions with confirmed cases.

o The maps help visualise mismatches between monitoring effort and predicted risk, indicating which areas are undersampled but high-risk.

o The maps in combination with human serosurveillance help assess population-level exposure and improve understanding on the infection status and circulation of TBEV.

o The maps inform national vaccination policies.

• Local:

o The maps support regional preparedness and enhance the likelihood of early recognition and differential diagnosis of TBE in humans; given the potential for introductions by migratory birds and the fact that every municipal health service region contained suitable areas for TBEV (R_0_ ≥ 1), all should be aware of TBEV, not only those with confirmed cases.

MHS: Municipal Health Service; R_0_: basic reproduction number; TBEV: tick-borne encephalitis virus.^a^ Figure 1 and Figure 2 Panels A, C, D and E.

The habitat-suitability metric (R_0_ ≥ 1) highlights areas of potential exposure to TBEV. Suitability was primarily driven by variation in the abundance of TBEV-competent hosts (small rodents) and the proportion of suitable habitat for the tick vector. Sites where TBEV was detected in ticks or rodents, and areas with seropositive roe deer, largely overlapped with those predicted as suitable for TBEV transmission. This association supported the accuracy of the model and suggests that this map could be used to identify areas in the Netherlands where targeted tick collection and testing for TBEV could be conducted to confirm local virus circulation, although the virus was also detected in areas with predicted R_0_ ≤ 1. While such values indicate that sustained circulation is unlikely, they do not exclude the possibility of short-lived circulation or isolated cases. Furthermore, most mismatches between observed data and predicted R_0_ < 1 involved roe deer seropositivity, this may be due to seropositivity reflecting past exposure rather than active infection [38,39]. Moreover, it is important to note that the input data may not be perfectly accurate, and the nature of the model as a simplification of reality (rather than a definitive truth) means some level of inexactitude is unavoidable.

Identifying potential microfoci is difficult as the risk is not uniform within a suitable grid cell. Currently, monitoring of TBEV in ticks is ongoing, with confirmation of TBEV in ticks at a location selected for targeted tick dragging based on a predicted R_0_ ≥ 1 and seropositive roe deer data. The inability to detect the virus at other sites up till now does not necessarily indicate its absence or misalignment with the model presented here. Rather, it could reflect the inherent limitations associated with field monitoring, including insufficient sample sizes and the relatively coarse spatial resolution of the model (1 km^2^) compared with the scale of TBEV microfoci (0.5–1 ha) [39]. Moreover, the modelling framework in this study represents habitat suitability, not the circulation (confirmed ongoing transmission) of this virus. Suitable means that all the biotic factors are available for circulation, but that the virus may or may not circulate there. For the latter, detection of virus circulation, our “golden standard” is the molecular detection of the virus in ticks. Moreover, as TBEV is still emerging in the Netherlands, the virus may not yet have expanded to fully occupy these ecological niches. In areas where ticks tested negative, but the model predicts suitability, targeted roe deer serosurveillance, ideally based on both ELISA and SNT, may provide a more sensitive means of detection than tick screening.

Shifts in host community composition may have contributed to the emergence and continued spread of TBEV within the Netherlands. Long-term trends in Dutch ecosystems suggest that certain mammal species, such as roe deer and bank vole, are increasing in abundance, while some bird populations are in decline [40-43]. These changes likely influence tick-host interactions and consequently TBEV transmission dynamics. For instance, a higher roe deer abundance increases the number of co-feeding tick groups on rodents [44]. A decline in bird abundance could further increase the probability of immature ticks feeding on competent rodent hosts, as they are less frequently diverted to birds [45]. In this study, we showed that host community composition influenced R_0_, with changes in the abundance of competent rodent species having the most pronounced effect. The inhibiting effect of incompetent hosts was limited, though birds had the greatest impact among them. The relatively small change in R_0_ might be attributable to such effects being more pronounced at local spatial scales. Alternatively, it could be related to how tick burden on hosts was parameterised in the model. Average tick burden per host was extracted from previous work [30]. We assumed tick burden per host was dependent on a tick’s ability to locate a host [30,46,47]. We therefore corrected the average tick burden per host using tick suitable habitat and density of hosts. As such, adding more hosts led to an increase in total tick counts and, in case of a higher density of competent hosts, to a higher R_0_. This approach did not consider that tick populations might be limited by factors other than host availability or the possibility of nonlinear relationships between host abundance and cofeeding. For instance, prior research showed that a higher density of rodents results in fewer ticks per rodent, thereby reducing the probability of co-feeding [39]. In our study, we assumed a fixed linear relationship between host density and tick burden, which may have resulted in an underestimation of the effect of shifts in host community and an overestimation of co-feeding.

High-resolution, contemporary data on mammal distributions and abundances remain scarce. We addressed this by integrating multiple data sources and modelling minimum and maximum estimates. While this approach increased spatial coverage, the underlying datasets have notable limitations, and their combination inevitably introduced uncertainty into the host density layer. Occurrence datasets, while broad and cost-effective due to citizen reporting, often lack structured sampling designs and rarely capture true absences [48]. They tend to disproportionately cover certain time periods, geographical regions or taxonomic groups that are sampled more often or more extensively than others [48-52]. This bias was particularly notable for rodents, where available occurrence data substantially underestimated distributions reported by the Dutch Mammal Society [52]. Hunting-bag data, while useful, suffered from variable reporting, lack of standardised effort metrics and regional inconsistencies. Their use a proxy for abundance was in consequence limited [53], and abundance estimates based on count data assume equal detectability across species and contexts [54], which was a condition not met in our study. Finally, climatic conditions were not explicitly included in the model; instead, we assumed that macroclimatic conditions in the Netherlands are generally suitable for *I. ricinus* and TBEV, and we represented abiotic variations solely via tick-habitat suitability.

## Conclusion

We developed and validated a spatial epidemiological R_0_-based habitat suitability framework that identifies areas where ecological conditions can sustain TBEV circulation, including in regions without confirmed presence based on OHM data. The resulting maps provide a basis for risk-based surveillance and control, and because this framework is adaptable it can continuously be refined as new host (particularly that of rodents), vector, and environmental data become available. It offers a tool to strengthen preparedness for TBEV and other emerging vector-borne pathogens.

## Data Availability

Data on the spatial abundance of bird species are available at Sovon (https://stats.sovon.nl/). Information on roe deer and fallow deer densities was extracted from fauna management plans. Data on mammalian occurrences are available at GBIF (https://www.gbif.org/). Hunting bag data (KNJV), count data (KNJV), and occurrence data (DMS) aggregated per Wildlife Management Unit are available from the corresponding author upon request. Data on tick burdens on hosts were retrieved from [2]. Observational data on TBEV in roe deer, rodents, and ticks are available in [3-6]. Additional observational data acquired in this study are available from the corresponding author upon request.

## Supplementary Data

1163000 Supplementary Material

## References

[1] Dekker M Laverman GD de Vries A Reimerink J Geeraedts F. Emergence of tick-borne encephalitis (TBE) in the Netherlands. Ticks Tick Borne Dis. 2019;10(1):176-9. 10.1016/j.ttbdis.2018.10.008 30385073

[2] Bogovic P Strle F. Tick-borne encephalitis: A review of epidemiology, clinical characteristics, and management. World J Clin Cases. 2015;3(5):430-41. 10.12998/wjcc.v3.i5.430 25984517 PMC4419106

[3] Bartholdsson S Hergens M-P Hansson KE Ragnarsson J Hodosi P Kus I Clinical characteristics of tick-borne encephalitis in adult patients: a 10-year retrospective study in Stockholm, Sweden. J Infect Dis. 2025;231(1):e195-205. 10.1093/infdis/jiae463 39316686 PMC11793045

[4] de Graaf JA Reimerink JH Voorn GP Bij de Vaate EA de Vries A Rockx B First human case of tick-borne encephalitis virus infection acquired in the Netherlands, July 2016. Euro Surveill. 2016;21(33):30318. 10.2807/1560-7917.ES.2016.21.33.30318 27562931 PMC4998423

[5] Weststrate AC Knapen D Laverman GD Schot B Prick JJ Spit SA Increasing evidence of tick-borne encephalitis (TBE) virus transmission, the Netherlands, June 2016. Euro Surveill. 2017;22(11):30482. 10.2807/1560-7917.ES.2017.22.11.30482 28333618 PMC5356422

[6] Jahfari S de Vries A Rijks JM Van Gucht S Vennema H Sprong H Tick-borne encephalitis virus in ticks and roe deer, the Netherlands. Emerg Infect Dis. 2017;23(6):1028-30. 10.3201/eid2306.161247 28518024 PMC5443429

[7] Esser HJ Lim SM de Vries A Sprong H Dekker DJ Pascoe EL Continued circulation of tick-borne encephalitis virus variants and detection of novel transmission foci, the Netherlands. Emerg Infect Dis. 2022;28(12):2416-24. 10.3201/eid2812.220552 36288572 PMC9707572

[8] Pascoe EL Bakker JW Wijburg SR de Vries A Sprong H Marcantonio M Multiple variants of tick-borne encephalitis virus in voles, mice and ticks, the Netherlands, 2021 to 2023. Euro Surveill. 2025;30(4):2400247. 10.2807/1560-7917.ES.2025.30.4.2400247 39885821 PMC11920782

[9] National Institute for Public Health and the Environment (RIVM). Tekenencefalitis |LCI-richtlijn. [Tick-borne encephalitis – recommendations]. Bilthoven: RIVM; 14 Jan 2025. Dutch. Available from: https://lci.rivm.nl/richtlijnen/tekenencefalitis

[10] Rijks JM Montizaan MGE Bakker N de Vries A Van Gucht S Swaan C Tick-borne encephalitis virus antibodies in roe deer, the Netherlands. Emerg Infect Dis. 2019;25(2):342-5. 10.3201/eid2502.181386 30666954 PMC6346459

[11] Dobler G, Hufert F, Pfeffer M, Essbauer S. Tick-borne encephalitis: From microfocus to human disease. In: Mehlhorn H (editor). Progress in parasitology. Dordrecht: Springer; 29 Jul 2011.p. 323-31. Available from: https://link.springer.com/chapter/10.1007/978-3-642-21396-0_17

[12] Estrada-Peña A de la Fuente J. The ecology of ticks and epidemiology of tick-borne viral diseases. Antiviral Res. 2014;108:104-28. 10.1016/j.antiviral.2014.05.016 24925264

[13] Randolph SE Miklisová D Lysy J Rogers DJ Labuda M. Incidence from coincidence: patterns of tick infestations on rodents facilitate transmission of tick-borne encephalitis virus. Parasitology. 1999;118(Pt 2):177-86. 10.1017/S0031182098003643 10028532

[14] Borde JP Kaier K Hehn P Matzarakis A Frey S Bestehorn M The complex interplay of climate, TBEV vector dynamics and TBEV infection rates in ticks–monitoring a natural TBEV focus in Germany, 2009-2018. PLoS One. 2021;16(1):e0244668. 10.1371/journal.pone.0244668 33411799 PMC7790265

[15] Valarcher JF Hägglund S Juremalm M Blomqvist G Renström L Zohari S Tick-borne encephalitis. Rev Sci Tech. 2015;34(2):453-66. 10.20506/rst.34.2.2371 26601448

[16] Jaenson TGT Petersson EH Jaenson DGE Kindberg J Pettersson JH-O Hjertqvist M The importance of wildlife in the ecology and epidemiology of the TBE virus in Sweden: incidence of human TBE correlates with abundance of deer and hares. Parasit Vectors. 2018;11(1):477. 10.1186/s13071-018-3057-4 30153856 PMC6114827

[17] Cagnacci F Bolzoni L Rosà R Carpi G Hauffe HC Valent M Effects of deer density on tick infestation of rodents and the hazard of tick-borne encephalitis. I: empirical assessment. Int J Parasitol. 2012;42(4):365-72. 10.1016/j.ijpara.2012.02.012 22464896

[18] Gerth H-J Grimshandl D Stage B Döller G Kunz C. Roe deer as sentinels for endemicity of tick-borne encephalitis virus. Epidemiol Infect. 1995;115(2):355-65. 10.1017/S0950268800058477 7589274 PMC2271401

[19] Morellet N Bonenfant C Börger L Ossi F Cagnacci F Heurich M Seasonality, weather and climate affect home range size in roe deer across a wide latitudinal gradient within Europe. J Anim Ecol. 2013;82(6):1326-39. 10.1111/1365-2656.12105 23855883

[20] Waldenström J Lundkvist A Falk KI Garpmo U Bergström S Lindegren G Migrating birds and tickborne encephalitis virus. Emerg Infect Dis. 2007;13(8):1215-8. 10.3201/eid1308.061416 17953095 PMC2828075

[21] Kelly PH Kwark R Marick HM Davis J Stark JH Madhava H Different environmental factors predict the occurrence of tick-borne encephalitis virus (TBEV) and reveal new potential risk areas across Europe via geospatial models. Int J Health Geogr. 2025;24(1):3. 10.1186/s12942-025-00388-9 40087786 PMC11908066

[22] Uusitalo R Siljander M Dub T Sane J Sormunen JJ Pellikka P Modelling habitat suitability for occurrence of human tick-borne encephalitis (TBE) cases in Finland. Ticks Tick Borne Dis. 2020;11(5):101457. 10.1016/j.ttbdis.2020.101457 32723626

[23] Hartemink NA Randolph SE Davis SA Heesterbeek JA. The basic reproduction number for complex disease systems: defining R(0) for tick-borne infections. Am Nat. 2008;171(6):743-54. 10.1086/587530 18462128

[24] Diekmann O, Heesterbeek JAP. Mathematical epidemiology of infectious diseases: model building, analysis and interpretation. Hoboken: John Wiley & Sons; 2000.

[25] Diekmann O Heesterbeek JAP Metz JAJ. On the definition and the computation of the basic reproduction ratio R0 in models for infectious diseases in heterogeneous populations. J Math Biol. 1990;28(4):365-82. 10.1007/BF00178324 2117040

[26] Benkimoun S Atyame C Haramboure M Degenne P Thébault H Dehecq J-S Dynamic mapping of dengue basic reproduction number. Results Phys. 2021;29:104687. 10.1016/j.rinp.2021.104687

[27] Hartemink NA Purse BV Meiswinkel R Brown HE de Koeijer A Elbers AR Mapping the basic reproduction number (R_0_) for vector-borne diseases: a case study on bluetongue virus. Epidemics. 2009;1(3):153-61. 10.1016/j.epidem.2009.05.004 21352762

[28] Hartemink N. Modelling and mapping the basic reproduction number R0 for canine leishmaniasis: a case study for a region in South West France. In: Hartemink N (PhD dissertation). Vector-borne diseases: the basic reproduction number R0 and risk maps. Utrecht: Utrecht university; 7 Dec 2009. Available from: https://research-portal.uu.nl/en/publications/vector-borne-diseases-the-basic-reproduction-number-r0-and-risk-m/

[29] Esser HJ Liefting Y Ibáñez-Justicia A van der Jeugd H van Turnhout CAM Stroo A Spatial risk analysis for the introduction and circulation of six arboviruses in the Netherlands. Parasit Vectors. 2020;13(1):464. 10.1186/s13071-020-04339-0 32912330 PMC7488554

[30] Fabri ND Heesterbeek H Cromsigt JPGM Ecke F Sprong H Nijhuis L Exploring the influence of host community composition on the outbreak potential of Anaplasma phagocytophilum and Borrelia burgdorferi s.l. Ticks Tick Borne Dis. 2024;15(1):102275. 10.1016/j.ttbdis.2023.102275 37922668

[31] Matser A Hartemink N Heesterbeek H Galvani A Davis S. Elasticity analysis in epidemiology: an application to tick-borne infections. Ecol Lett. 2009;12(12):1298-305. 10.1111/j.1461-0248.2009.01378.x 19740112

[32] Danielová V Holubová J Pejcoch M Daniel M. Potential significance of transovarial transmission in the circulation of tick-borne encephalitis virus. Folia Parasitol (Praha). 2002;49(4):323-5. 10.14411/fp.2002.060 12641208

[33] Militzer N Bartel A Clausen P-H Hoffmann-Köhler P Nijhof AM. Artificial feeding of all consecutive life stages of Ixodes ricinus. Vaccines (Basel). 2021;9(4):385. 10.3390/vaccines9040385 33919961 PMC8070929

[34] Streng K Hakze-van der Honing RW Graham H van Oort S de Best PA Abourashed A Orthoflavivirus surveillance in the Netherlands: Insights from a serosurvey in horses & dogs and a questionnaire among horse owners. Zoonoses Public Health. 2024;71(8):900-10. 10.1111/zph.13171 39057842

[35] Schwaiger M Cassinotti P. Development of a quantitative real-time RT-PCR assay with internal control for the laboratory detection of tick borne encephalitis virus (TBEV) RNA. J Clin Virol. 2003;27(2):136-45. 10.1016/S1386-6532(02)00168-3 12829035

[36] Hassall RM Holding M Medlock JM Asaaga FA Vanwambeke SO Hewson R Identifying hotspots and risk factors for tick-borne encephalitis virus emergence at its range margins to guide interventions, Great Britain. Euro Surveill. 2025;30(13):2400441. 10.2807/1560-7917.ES.2025.30.13.2400441 40183125 PMC11969960

[37] de Jongh L, de Jong R, Schenau S, van Berkel J, Bogaart P, Driessen C, et al. Natuurlijk Kapitaalrekeningen Nederland 2013-2018. [Natural Capital Accounts the Netherlands 2013-2018]. The Hague: Statistics Netherlands; 30 Jul 2021. Dutch. Available from: https://www.cbs.nl/nl-nl/longread/aanvullende-statistische-diensten/2021/natuurlijk-kapitaalrekeningen-nederland-2013-2018

[38] Ollivier V Choquet R Gamble A Bastien M Combes B Gilot-Fromont E Temporal dynamics of antibody level against Lyme disease bacteria in roe deer: Tale of a sentinel? Ecol Evol. 2023;13(8):e10414. 10.1002/ece3.10414 37600488 PMC10433119

[39] Rosà R Tagliapietra V Manica M Arnoldi D Hauffe HC Rossi C Changes in host densities and co-feeding pattern efficiently predict tick-borne encephalitis hazard in an endemic focus in northern Italy. Int J Parasitol. 2019;49(10):779-87. 10.1016/j.ijpara.2019.05.006 31348960

[40] Statistics Netherlands (CBS). Toename meeste zoogdieren sinds 1995, afname onder beschermde soorten na 2011. [Number of mammals increased since 1995, protected species decreased after 2011]. The Hague: CBS; 30 Oct 2023. Dutch. Available from: https://www.cbs.nl/nl-nl/nieuws/2023/44/toename-meeste-zoogdieren-sinds-1995-afname-onder-beschermde-soorten-na-2011

[41] Koninklijke Nederlandse Jagersvereniging (KNJV). Reeën. [Roe deer]. Amersfoort: KNJV. [Accessed: 18 May 2026]. Dutch. Available from: https://www.jagersvereniging.nl/onderwerp/standpunt-reeen

[42] van den Bremer L, van Turnhout C. Voorstudie Jaar van de Merel 2022. Sovon-rapport 2021/56. [Preliminary study year of the Blackbird 2022]. Nijmegen: Sovon Vogelonderzoek Nederland; 2021. Dutch. Available from: https://stats.sovon.nl/static/publicaties/rap_2021-56_jaar-vd-merel-2021.pdf

[43] van Beusekom R. Vogelbalans 2021: hoe vergaat het onze vogels? [Bird counting: how are our birds doing?]. Zeist: Vogelbescherming; 10 Jan 2022. Dutch. Available from: https://www.vogelbescherming.nl/actueel/bericht/vogelbalans-2021-hoe-vergaat-het-onze-vogels

[44] Rizzoli A Hauffe HC Tagliapietra V Neteler M Rosà R. Forest structure and roe deer abundance predict tick-borne encephalitis risk in Italy. PLoS One. 2009;4(2):e4336. 10.1371/journal.pone.0004336 19183811 PMC2629566

[45] Dagostin F Tagliapietra V Marini G Ferrari G Cervellini M Wint W High habitat richness reduces the risk of tick-borne encephalitis in Europe: A multi-scale study. One Health. 2023;18:100669. 10.1016/j.onehlt.2023.100669 38283833 PMC10820641

[46] Randolph S, Chemini C, Furlanello C, Genchi C, Hails R. The ecology of tick-borne diseases in wildlife reservoirs. In: Hudson P, Rizzoli A, Grenfell B, Heesterbeek H, Dobson A (editors). The Ecology of Wildlife Diseases. Oxford: Oxford University Press; 3 Jan 2002. p. 119-38.

[47] Jaenson TG Hjertqvist M Bergström T Lundkvist A. Why is tick-borne encephalitis increasing? A review of the key factors causing the increasing incidence of human TBE in Sweden. Parasit Vectors. 2012;5(1):184. 10.1186/1756-3305-5-184 22937961 PMC3439267

[48] Farr MT Green DS Holekamp KE Zipkin EF. Integrating distance sampling and presence-only data to estimate species abundance. Ecology. 2021;102(1):e03204. 10.1002/ecy.3204 32970847

[49] Bird TJ Bates AE Lefcheck JS Hill NA Thomson RJ Edgar GJ Statistical solutions for error and bias in global citizen science datasets. Biol Conserv. 2014;173:144-54. 10.1016/j.biocon.2013.07.037

[50] Dickinson JL Zuckerberg B Bonter DN. Citizen science as an ecological research tool: challenges and benefits. Annu Rev Ecol Evol Syst. 2010;41(1):149-72. 10.1146/annurev-ecolsys-102209-144636

[51] Phillips SJ Dudík M Elith J Graham CH Lehmann A Leathwick J Sample selection bias and presence-only distribution models: implications for background and pseudo-absence data. Ecol Appl. 2009;19(1):181-97. 10.1890/07-2153.1 19323182

[52] Zoogdiervereniging. Bosmuis. [Wood mouse]. Nijmegen: Zoogdiervereniging. [Accessed: 18 May 2026]. Dutch. Available from: https://www.zoogdiervereniging.nl/zoogdiersoorten/bosmuis

[53] Forsyth DM Comte S Davis NE Bengsen AJ Côté SD Hewitt DG Methodology matters when estimating deer abundance: a global systematic review and recommendations for improvements. J Wildl Manage. 2022;86(4):e22207. 10.1002/jwmg.22207

[54] Royle JA Nichols JD. Estimating abundance from repeated presence–absence data or point counts. Ecology. 2003;84(3):777-90. 10.1890/0012-9658(2003)084[0777:EAFRPA]2.0.CO;2

[55] Informatiepunt Leefomgeving (IPLO). Faunabeheerplan en faunabeheereenheid. [Wildlife management plan and management unit]. Hague: IPLO. [Accessed: 18 May 2026]. Dutch. Available from: https://iplo.nl/thema/natuur/faunabeheerplan-faunabeheereenheid

