# Supplementary material for: Targeted monitoring informed by mapping the ongoing spread of tick-borne encephalitis virus, the Netherlands
Source: Euro Surveill. 2026 May 21;31(20):2500767. doi: 10.2807/1560-7917.ES.2026.31.20.2500767 (PMC13197735; doi:10.2807/1560-7917.ES.2026.31.20.2500767)
Supplement: Supplementary Material [file 25-00767_WIJBURG_Supplement.pdf]

Supplementary files

This supplementary material is hosted by *Eurosurveillance* as supporting information alongside the article *Targeted Monitoring Informed by Mapping the Ongoing Spread of TBEV Across the Netherlands*, on behalf of the authors, who remain responsible for the accuracy and appropriateness of the content. The same standards for ethics, copyright, attributions, and permissions as for the article apply. Supplements are not edited by *Eurosurveillance,* and the journal is not responsible for the maintenance of any links or email addresses provided therein.

Overview Supplementary Files

Supplement S1. Host community per km^2

- Supplementary Text S1. Host community per km^2
  - Raw Data – Suitable Host Habitat
  - Raw Data – Hunting-Based Data & Large Mammal Count Data
  - Raw Data – Occurrence Data
  - Raw Data – Fauna Management Plans for Large Mammal Species
  - Processed Data – Large Mammal Abundance Estimation
  - Processed Data – Small Mammal Abundance Estimation
  - Processed Data – Abundance Bird Species
- Supplementary Tables S1. Host community per km^2
- Supplementary Figures S1. Host community per km^2

Supplement S2. Suitable Tick Habitat

- Supplementary Text S2. Suitable Tick Habitat
- Supplementary Tables S2. Suitable Tick Habitat
- Supplementary Figures S2. Suitable Tick Habitat

Supplement S3. Epidemiological modelling and Scenarios

- Supplementary Text S3. Epidemiological modelling and Scenarios
- Supplementary Tables S3. Epidemiological modelling and Scenarios
- Supplementary Figures S3. Epidemiological modelling and Scenarios

Supplement S4. Model Validation

- Supplementary Text S4. Model Validation
- Supplementary Tables S4. Model Validation

Supplement S5. Targeted Monitoring

- Supplementary Text S5. TBEV Expansion Assessment Based on Roe Deer Samples
- Supplementary Text S5. Targeted Monitoring in Ticks
- Supplementary Figure S5. Targeted Monitoring in Ticks

Supplement S1. Host community per km^2

Supplementary Text S1. Host community per km^2

Host community included *Apodemus* spp., *Clethrionomys glareolus*, *Microtus agrestis*, roe deer (*Capreolus capreolus*), common fallow deer (*Dama dama*), red fox (*Vulpes vulpes*), and several bird species (i.e., European stonechat (*Saxicola rubicola*), Common blackbird (*Turdus merula*), Tree pipit (*Anthus trivialis*), Eurasian skylark (*Alauda arvensis*), Song thrush (*Turdus philomelos*), Dunnock (*Prunella modularis*), Eurasian jay (*Garrulus glandarius*), Common starling (*Sturnus vulgaris*), Hawfinch (*Coccothraustes coccothraustes*), European robin (*Erithacus rubecula*), and Common chaffinch (*Fringilla coelebs*)).

Raw Data – Suitable Host Habitat

The habitat types suitable for the different mammalian host species were identified using the global habitat map (~100m resolution) [1]. This map consisted of 47 habitat classes as defined by the IUCN (International Union for Conservation of Nature and Natural Resources) Red List [1]. The global habitat map was cropped to the spatial extent of the Netherlands; hereafter 18 habitat types remained (Supplementary Table S1). The IUCN classifies habitat types as either of major importance (3), suitable (2) or marginal (1) for a species [2-8]. All other habitat types were classified as unsuitable (0) for that specific species. Habitat suitability for each host species was calculated as the proportion of suitable and marginal area in a km^2 grid cell.

Raw Data – Hunting-Based Data & Large Mammal Count Data

Hunting-based data on the number of roe deer (*Capreolus capreolus*), common fallow deer (*Dama dama*), and the red fox (*Vulpes vulpes*) culled in an area (i.e., hunting bag statistics) were obtained from the Royal Dutch Hunters' Association (KNJV) - www.Faunaregistratie.nl for the years 2014 till 2024 (resolution 1 km^2; Supplementary Table S2). Roe deer and common fallow deer counts for the years 2015 to 2024 were additionally provided by the KNJV (resolution 1 km^2; Supplementary Table S2). Roe deer and common fallow deer counts represented the minimal number alive in a given area; the number per grid cell was based on three counting sessions in Spring. Data on hunting bag statistics and counts were unavailable for all years for the province of South Holland because of a different registration system. Additionally, after 2023, no detailed hunting-based data were available for North Holland due to a change in registration systems. For both data sources, yearly averages ± standard deviation (SD) were calculated per squared kilometer grid cell; yearly trends have been shown in Supplementary Figure S1A. Additionally, the correlation between the two datasets was analyzed (see description Supplementary Figure S1).

Raw Data – Occurrence Data

Occurrence (i.e., presence-only) data on roe deer, fallow deer, and red fox were obtained from the Dutch Mammal Society (DMS). This dataset contained occurrences reported by citizens (available at: https://observation.org/), as well as occurrences documented via Ecological Monitoring Network measurement programs. Data were available from 2010 till 2021 (resolution 1 km^2). We additionally downloaded occurrence data on roe deer (233,681 unique sightings in 2,778 grid cells), common fallow deer (15,638 unique sightings in 778 grid cells), red fox (58,758 unique sightings in 2,109 grid cells), bank vole (*Clethrionomys glareolus*, 10,917 unique sightings in 1,420 grid cells), short-tailed field vole (*Microtus agrestis*, 145 unique sightings in 119 grid cells), yellow-necked field mouse (*Apodemus flavicollis*, 790 unique sightings in 157 grid cells), and wood mouse (*Apodemus sylvaticus*, 18,142 unique sightings in 1,754 grid cells) from Global Biodiversity Information Facility platform (GBIF, available at: https://www.gbif.org; 2010 – 2025) [9-15]. Data obtained from GBIF were transformed from point-location data to gridded raster data (resolution 1 km^2).

Raw Data – Fauna Management Plans for Large Mammal Species

Information on roe deer and common fallow deer densities per wildlife management unit (WMU, administratively defined areas within provinces that are used for practical wildlife management; N=285) or per NUTS2 region (i.e., provinces, main administrative units in the

Netherlands; N=12) was extracted from fauna management plans (FMPs; Supplemental Figure S1B; [16-31]). Where possible, data were collected for the years 2014 to 2024.

Processed Data – Large Mammal Abundance Estimation

To redistribute density estimates of roe deer (available at WMU or NUTS2 scale), common fallow deer (available at WMU or NUTS2 scale), and red foxes (available at national scale [32]) to a resolution of 1 km^2, cells were assigned a score based on the datasets available for that species. Taking roe deer as an example, within a WMU or NUTS2 region (*j*), each grid cell (*i*) was evaluated based on four factors: proportion of suitable habitat types, hunting bag statistics, count data, and occurrence data. Per grid cell (*i*) a score per factor (*f*) was calculated ($score = Factor_{f,i}/\sum_j Factor_{f,j}$). The calculated scores for the different factors (*f*) were then summed to assign a combined score per grid cell (*i*). We used these scores to rank cells against each other within the same WMU or NUTS2 region. Cells with higher scores, indicating greater importance/suitability, were assigned a larger share of the total value. A score of zero was assigned to cells where a species was known to be absent [33]. Values for the proportion of suitable habitat, hunting bag statistics, counts, and occurrences were standardized within an area (i.e., national, NUTS2, WMU) using relative proportion standardization. Final density estimates have been provided in Supplementary Figure S2A – C.

Processed Data – Small Mammal Abundance Estimation

The bank vole, short-tailed field vole, yellow-necked field mouse, and wood mouse tended to be observed less frequently than the larger species. Consequently, we used Maximum Entropy Modelling (MaxEnt), a presence-only species distribution modelling approach, to estimate the relative likelihood of presence of these species [34, 35]. We used the R package 'dismo' version 1.3-16 to run the MaxEnt model [36]. As environmental predictors in our MaxEnt model, we included the standard 19 bioclimatic variables (30 arc-second resolution (~1km^2), Supplementary Table S3) from WorldClim version 2 (based on data from 1970 to 2000) [37]. Preprocessing of occurrence data included removal of records with missing or incorrect coordinates, removal of duplicate coordinates, and thinning the dataset to allow one occurrence datapoint per 1 km^2 grid cell. We then generated background points by randomly sampling spatial points across the Netherlands. Seventy percent of the occurrence data was used to train the model and 30% to test the performance. The final model was used to predict the relative likelihood of the presence of the bank vole, short-tailed field vole, yellow-necked field mouse, and wood mouse across the Netherlands.

No density estimates were available for the bank vole, short-tailed field vole, yellow-necked field mouse, and wood mouse. Hofmeester et al. [38] reports an average density of 1,200 bank voles, 1,000 short-tailed field voles, 1,200 yellow-necked field mice, and 1,200 wood mice per km^2 in forested areas. We assumed similar densities in areas as suitable as forests according to the IUCN (see *Raw data – Habitat suitability for hosts*). To estimate the density of these species per km^2, we took the product of the proportion of suitable habitat within each grid cell, the probability of presence predicted by MaxEnt, and the density estimates provided by [38] (Supplementary Figure S3A – D).

Processed Data – Abundance Bird Species

Eleven bird species were selected based on expert consultation. Depending on the bird species, breeding densities were available either on 1 km^2 grid cells or on 25 km^2 grid cells (Supplementary Table S3). All bird datasets were resampled to a resolution of 1 km^2. Because Sovon presents bird densities as nine categorical scores ranging from low to high, categorical scores were transformed to numerical abundance estimates by using the mean (Supplementary Figure S2D), minimum, and maximum breeding population ranges (Supplementary Table S3). The influence of bird migration on bird abundance was not considered. For more details on fieldwork methods and bird abundance modelling techniques, see Sovon [39].

Supplementary Tables S1. Host community per km^2

Table S1. Habitat classes in the Netherlands according to the IUCN habitat cover categorization.

IUCN-class	%
Artificial/Terrestrial – Arable Land	49.15
Forest – Temperate	4.11
Grassland	0.01
Grassland – Temperate	0.18
Artificial/Terrestrial – Pastureland	10.42
Artificial/Terrestrial – Plantations	11.83
Shrubland – Temperate	13.16
Artificial/Terrestrial – Urban areas	8.05
Wetlands (inland)	2.11
Wetlands (inland) – Permanent freshwater lakes (over 8 ha)	0.59
Wetlands (inland) – Permanent freshwater marshes/pools (under 8 ha)	0.03
Wetlands (inland) – Permanent rivers/streams/creeks (includes waterfalls)	0.28
Wetlands (inland) – Permanent saline, brackish or alkaline lakes	0.00
Wetlands (inland) – Seasonal/intermittent freshwater lakes (over 8 ha)	0.01
Wetlands (inland) – Seasonal/intermittent saline, brackish or alkaline lakes and flats	0.04
Wetlands (inland) – Seasonal/intermittent/irregular rivers/streams/creeks	0.04

Table S2. Data layers used per wildlife species to calculate abundance per grid cell.

Parameter	Wildlife species	Layers used in the calculation of abundance	Reference value/layer
D_{rd}<br>D_{fd}	Roe deer<br>Fallow deer	Proportion of suitable habitat maps (1 km^2 resolution)<br>Hunting bag data (1 km^2 resolution)<br>Count data (KNJV; 1 km^2 resolution)<br>Occurrence data (1 km^2 resolution)	Density data (wildlife management plan; NUTS2 or WMU level) [a-p]
D_{rf}	Red fox	Proportion of suitable habitat (1 km^2 resolution)<br>Hunting bag data (1 km^2 resolution)<br>Occurrence data (1 km^2 resolution)	~120,000 (national level) [q]
D_{bv}	Bank vole	Proportion of suitable habitat (1 km^2 resolution)<br>Occurrence data (1 km^2 resolution)<br>Bioclimatic parameters (~1km^2)	1200 km^{-2} in forest areas [r]
D_{stfv}	Short-tailed field vole		1000 km^{-2} in forest areas [r]
D_{ynfm}	Yellow-necked field mouse		1200 km^{-2} in forest areas [r]
D_{wm}	Wood mouse		1200 km^{-2} in forest areas [r]
D_{es}	European stonechat *(Saxicola rubicola)* [a1]	Breeding density maps (1 km^2 resolution or 25 km^2 resolution)	Breeding population: 18,000-22,000 [s]
D_{cb}	Common blackbird *(Turdus merula)*		Breeding population: 500,000-900,000 [t]
D_{tp}	Tree pipit (*Anthus trivialis*) [a1]		Breeding population: 44,000-72,000 [u]
D_{esk}	Eurasian skylark (*Alauda arvensis*)		Breeding population: 34,000-44,000 [v]
D_{st}	Song thrush (*Turdus philomelos*)		Breeding population: 100,000-170,000 [w]
D_{d}	Dunnock (*Prunella modularis*)		Breeding population: 175,000-225,000 [x]
D_{ej}	Eurasian jay (*Garrulus glandarius*) [a1]		Breeding population: 47,000-68,000 [y]
D_{cs}	Common starling (*Sturnus vulgaris*)		Breeding population: 400,000-700,000 [z]
D_{h}	Hawfinch (*Coccothraustes coccothraustes*) [a1]		Breeding population: 16,000-20,000 [aa]
D_{er}	European robin (*Erithacus rubecula*)		Breeding population: 300,000-430,000 [ab]
D_{cc}	Common chaffinch (*Fringilla coelebs*)		Breeding population: 400,000-500,000 [ac]

[a1] Available at 5x5 km^2 resolution.
Subscripts b-ac correspond to the following sources: a-p = [16-31], q = [32], r = [38], s = [40], t = [41], u = [42], v = [43], w = [44] , x = [45], y = [46], z = [47], aa = [48], ab = [49], and ac = [50].

Table S3. Bioclimatic variables included in the MaxEnt model. Data were obtained from (https://www.worldclim.org/data/worldclim21.html, Accessed on 9th of April 2025).

Code	Description
BIO1	Annual mean temperature
BIO2	Mean diurnal range (Mean of monthly (max temp - min temp))
BIO3	Isothermality (BIO2/BIO7) (×100)
BIO4	Temperature Seasonality (standard deviation ×100)
BIO5	Max Temperature of Warmest Month
BIO6	Min Temperature of Coldest Month
BIO7	Temperature Annual Range (BIO5-BIO6)
BIO8	Mean Temperature of Wettest Quarter
BIO9	Mean Temperature of Driest Quarter
BIO10	Mean Temperature of Warmest Quarter
BIO11	Mean Temperature of Coldest Quarter
BIO12	Annual Precipitation
BIO13	Precipitation of Wettest Month
BIO14	Precipitation of Driest Month
BIO15	Precipitation Seasonality (Coefficient of Variation)
BIO16	Precipitation of Wettest Quarter
BIO17	Precipitation of Driest Quarter
BIO18	Precipitation of Warmest Quarter
BIO19	Precipitation of Coldest Quarter

Supplementary Figures S1. Host community per km^2

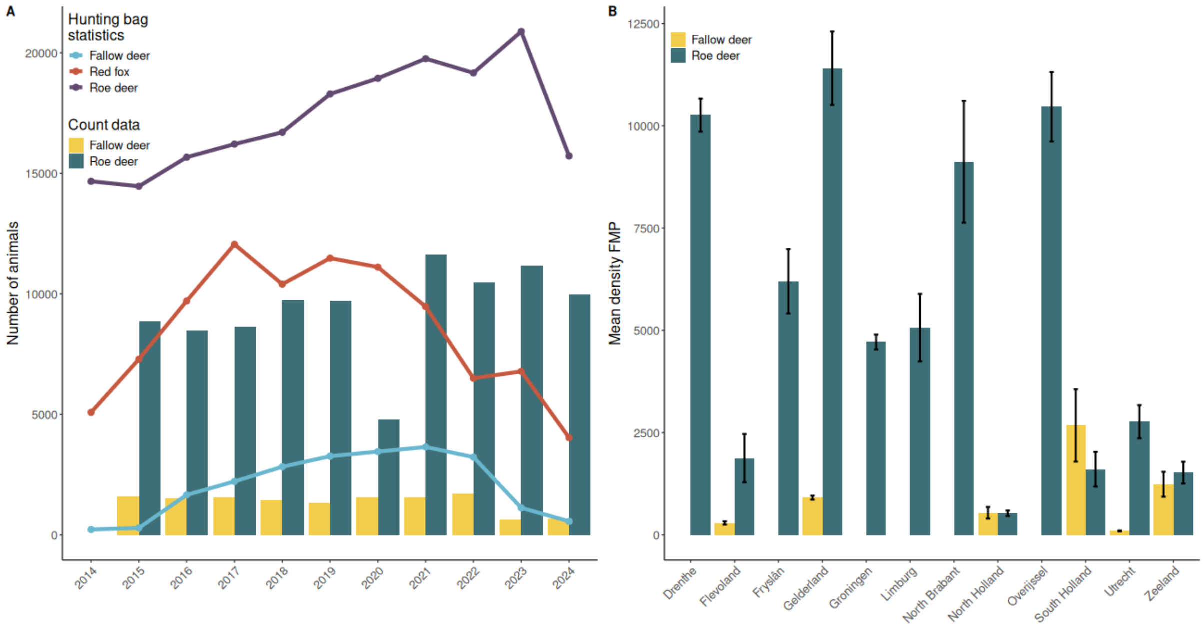

Figure S1 (A-B). Panel A. Data on hunting bag statistics (roe deer (*Capreolus capreolus*, N grid cells = 16,612), fallow deer (*Dama dama*, N grid cells = 612), and red fox (*Vulpes vulpes*, N grid cells = 14,991)) and count data (roe deer (N grid cells = 8,479) and fallow deer (N grid cells = 1,061)) provided by the Royal Dutch Hunters' Association (KNJV). **Panel B.** Bar plot displaying the average roe deer and fallow deer density per NUTS2 region (extracted from Wildlife Management Plans (WMP)), error bars indicate the standard deviation (SD). Roe deer counts showed a weak positive correlation with hunting-based data (Pearson correlation coefficient, $r(6{,}489) = 0.23$, $p < 0.0001$), in contrast to common fallow deer counts, which were more strongly positively correlated with hunting-based data, $r(132) = 0.80$, $p < 0.0001$.

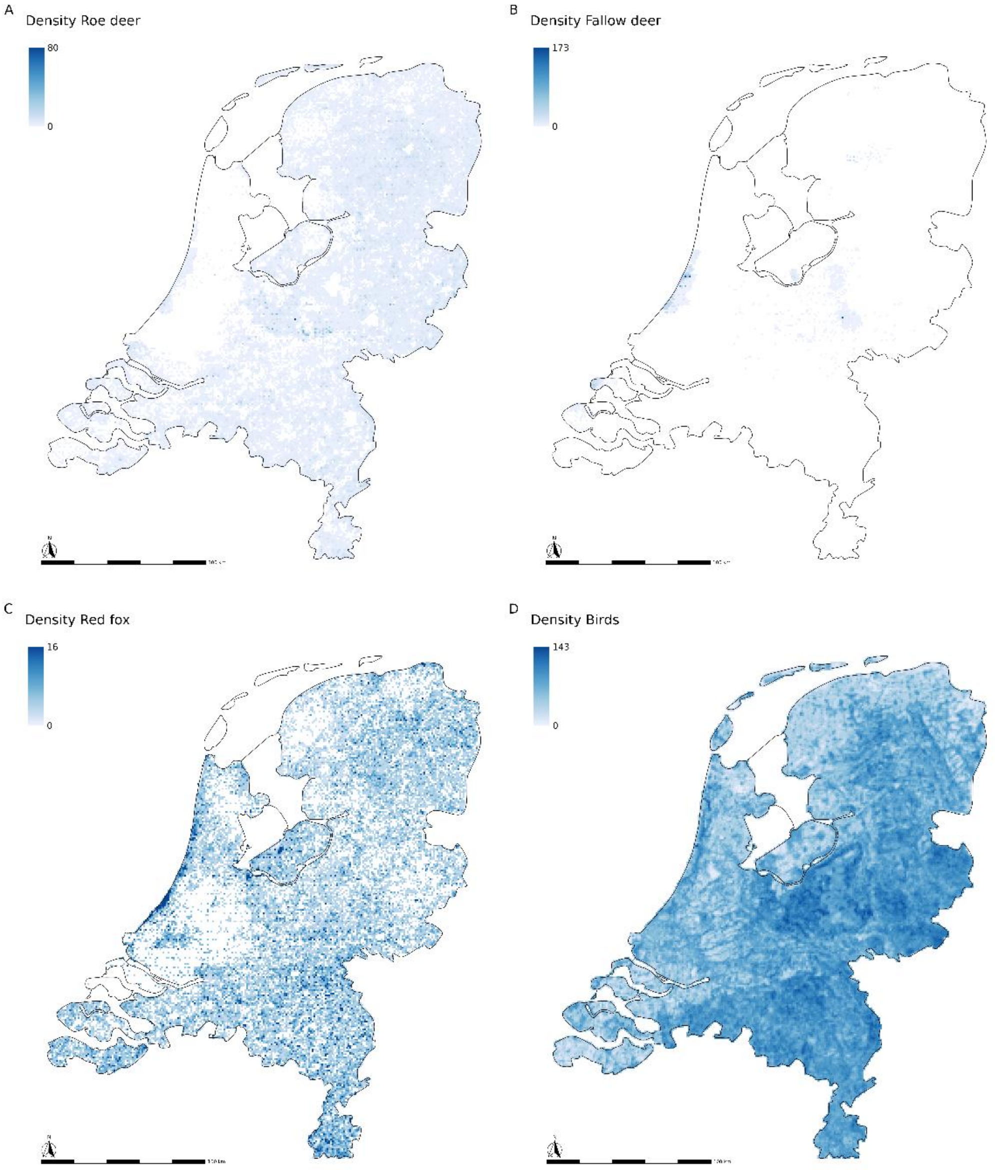

Figure. S2. Estimates density maps of roe deer (*Capreolus capreolus*; **Panel A**), common fallow deer (*Dama dama*; **Panel B**), red foxes (*Vulpes vulpes*; **Panel C**), and birds (**Panel D**).

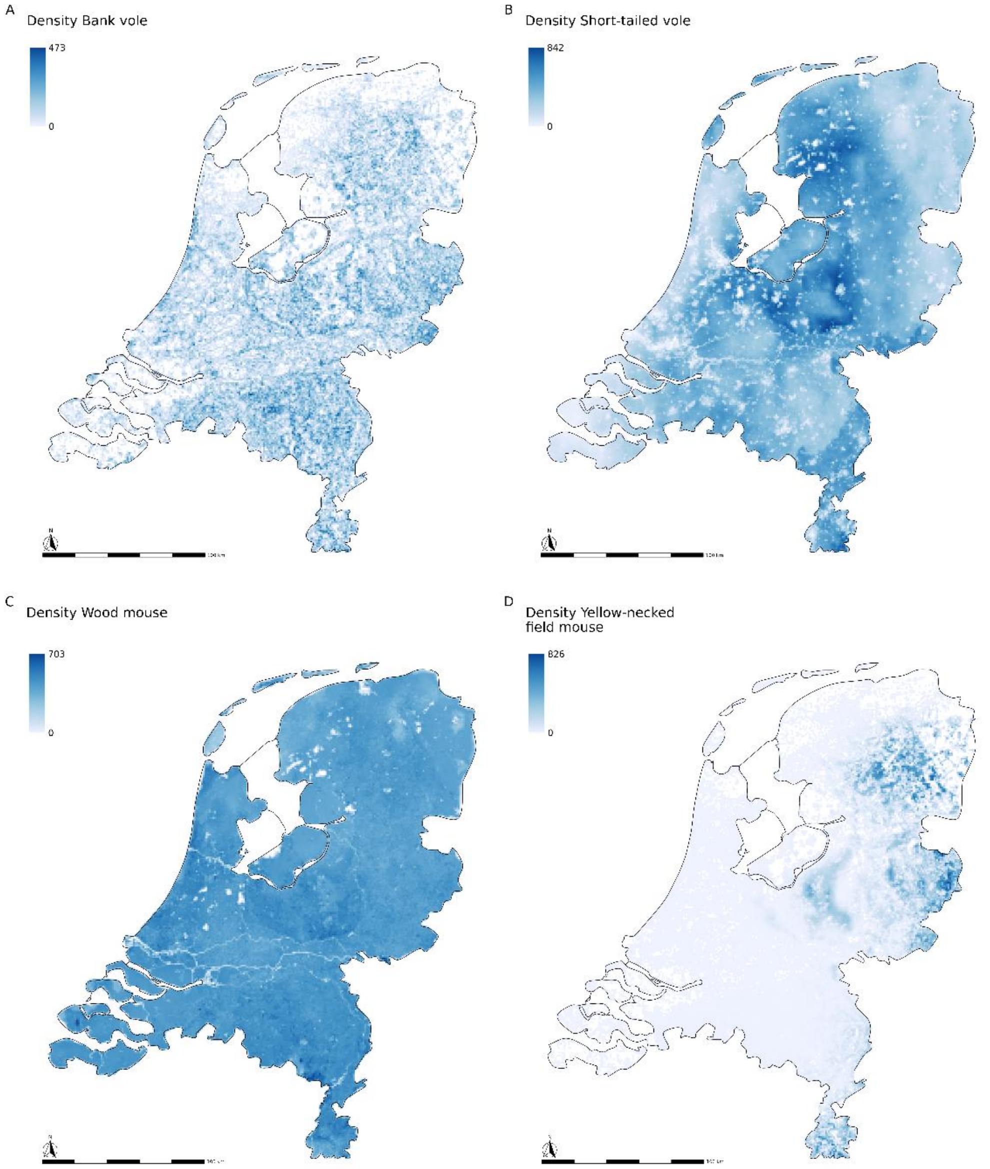

Figure. S3. Estimated density maps of the bank vole (*Clethrionomys glareolus*; **Panel A**), the short-tailed vole (*Microtus agrestis*; **Panel B**), the wood mouse (*Apodemus sylvaticus*; **Panel C**), and the yellow-necked field mouse (*A. flavicollis*; **Panel D**).

Supplement S2. Suitable Tick Habitat

Supplementary Text S2. Suitable Tick Habitat

Ixodes ricinus ticks occur in various habitat types but have a preference for woodland areas and forests with dense undergrowth [51, 52]. To account for these differences in preference, land-use types present in The Netherlands were classified as suitable (1) or as unsuitable (0) for *I. ricinus* ticks using methods described by Esser et al. 2020 (Supplementary Table S4) [53, 54]. We used the national land-use database (LGN2021), which has a spatial resolution of five meters [55]. In the LGN2021 database, 51 land use types have been defined, with key categories including agricultural crops, forest, water, nature, infrastructure, and urban areas. Per grid cell the proportion of suitable habitat was calculated. Spatial differences regarding the proportion of land-use types suitable for *I. ricinus* ticks are displayed in Supplementary Figure S4. Information on the proportion of suitable habitat per grid cell was used to adjust the average number of ticks per host within a grid cell.

Supplementary Tables S2. Suitable Tick Habitat

Table S4. Tick suitable habitats classification based on National land-use database (LGN2021) land use categories.

LGN2021 ID	LGN2021 name Dutch	LGN2021 name English	Unsuitable (0)/ Suitable (1)
1	Agrarisch gras	Pasture	0
2	Mais	Maise	0
3	Aardappelen	Potatoes	0
4	Bieten	Beets	0
5	Granen	Cereals	0
6	Overige landbouwgewassen	Other agricultural crops	0
8	Glastuinbouw	Greenhouses	0
9	Boomgaarden	Orchards	0
10	Bloembollen	Flower bulbs	0
11	Loofbos	Deciduous forest	1
12	Naaldbos	Coniferous forest	1
16	Zoet water	Fresh water	0
17	Zout water	Salt water	0
18	Bebouwing in primair bebouwd gebied	Urban built-up areas	0
19	Bebouwing in secundair bebouwd gebied	Semi-urban built-up areas	0
20	Bos in primair bebouwd gebied	Forest in built-up areas	1
22	Bos in secundair bebouwd gebied	Forest in semi-built-up areas	1
23	Gras in primair bebouwd gebied	Grass in built-up areas	0
24	Kale grond in bebouwd gebied	Bare ground in built-up areas	0
26	Bebouwing in buitengebied	Built-up in rural areas	0
27	Overig grondgebruik in buitengebied	Other land use in agricultural areas	0
28	Gras in secundair bebouwd gebied	Grass in semi built-up areas	1
29	Zonneparken	Solar parks	0
30	Kwelders	Salt marches	0
31	Open zand in kustgebied	Coastal sands	0
32	Duinen met een lage vegetatie	Dunes with low vegetation	1
33	Duinen met een hoge vegetatie	Dunes with high vegetation	1
34	Duinheide	Heathland in coastal areas	1
35	Open stuifzand en/of rivierzand	Drifting sands/river sandbanks	0
36	Heide	Heath	1
37	Matig vergraste heide	Grassy heathland	1
38	Sterk vergraste heide	Very grassy heathland	1
39	Hoogveen	Raised bogs	1
40	Bos in hoogveengebied	Forest in raised bogs	1
41	Overige moeras vegetatie	Other swamp vegetation	0
42	Rietvegetatie	Reeds	0
43	Bos in moerasgebied	Forest in swamp areas	1
45	Natuurgraslanden	Natural grasslands	1
46	Gras in kustgebied	Grasses in coastal areas	1
47	Overige gras	Other grasses	1
61	Boomkwekerijen	Tree nurseries	0
62	Fruitkwekerijen	Fruit cultivation	0
251	Hoofdinfrastructuur en spoorbaanlichamen	Roads	0
252	Halfverharde wegen, infrastructuur langzaam verkeer en overige infrastructuur	Half-hard infrastructure roads	0
253	Smalle wegen	Narrow roads	0
321	Struikvegetatie in hoogveengebied (laag)	Bush/shrub vegetation in raised bogs (low)	1
322	Struikvegetatie in moerasgebied (laag)	Bush/shrub vegetation in swamp areas	1
323	Overige struikvegetatie (laag)	Other bush/shrub vegetation (low)	1
331	Struikvegetatie in hoogveengebied (hoog)	Bush/shrub vegetation in raised bogs (high)	1

332	Struikvegetatie in moerasgebied (hoog)	Bush/shrub vegetation in swamp areas (high)	1
333	Overige struikvegetatie (hoog)	Other bush/shrub vegetation (high)	1

Supplementary Figures S2. Suitable Tick Habitat

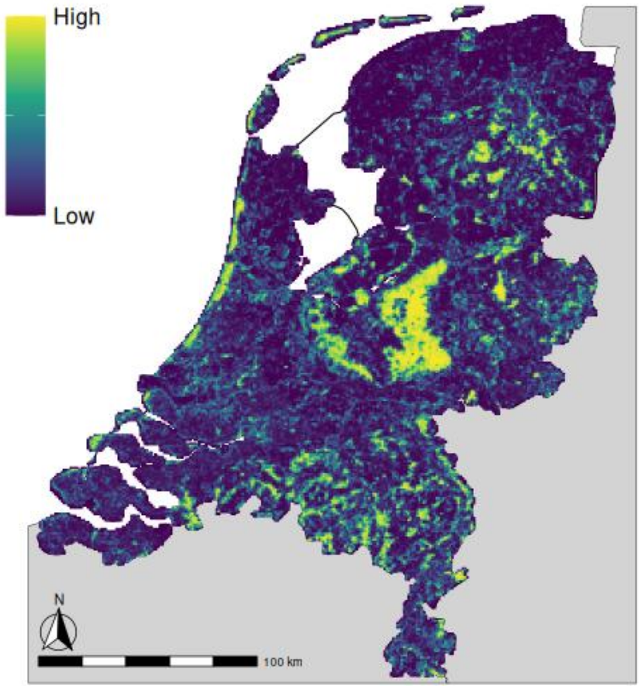

Figure. S4. Tick habitat suitability classification based on LGN2021 land use categories (habitat suitability ranged from 0 – 1). Suitability categorization has been provided in **Table S4**.

Supplement S3. Epidemiological modelling and scenarios

Supplementary Text S3. Epidemiological modelling and scenarios

The basic reproduction number was calculated using the Next generation matrix (NGM); a detailed description regarding the derivation of the NGM has been provided in [56, 57].

We used a five-dimensional next generation matrix (NGM) to model the R_0 for TBEV. The NGM contained the following types-at-infection (i.e., the expected number of infected individuals of type *i* caused by a single infectious individual type *j* [58]): ticks infected as eggs (E), ticks infected as larvae (L), ticks infected as nymphs (N), ticks infected as adults (A), and infected rodents (R; i.e., bank vole, the short-tailed field vole, the yellow-necked field mouse, and the wood mouse [59]). Dead-end hosts, i.e., non-competent hosts (e.g., roe deer, birds), were not considered as a type-at-infection as they do not contribute directly to the transmission cycle of TBEV [60], they were, however, included in equations 1 – 3 as they affect parameters such as tick survival probabilities and the probability of ticks feeding on competent hosts.

The elements *k11*, *k12*, *k13*, and *k14* represented the number of infected ticks at birth (i.e., transovarial transmission [61, 62]), the elements *k21*, *k22*, *k23*, *k31*, *k32*, *k33*, *k41*, *k42*, and *k43* represented transmission via co-feeding (i.e., non-systemic transmission [63-66]), the elements *k51*, *k52*, and *k51* represented transmission from an infected tick to a host (i.e., systemic transmission), and the elements *k25*, *k35*, and *k45* represented transmission from an infected host to an uninfected tick (i.e., systemic transmission [67]) [56]. Transmission via sexual interaction was not considered [68].

$$K = \begin{bmatrix} & E & L & N & A & R \\ E & k11 & k12 & k13 & k14 & 0 \\ L & k21 & k22 & k23 & 0 & k25 \\ N & k31 & k32 & k33 & 0 & k35 \\ A & k41 & k42 & k43 & 0 & k45 \\ R & k51 & k52 & k53 & 0 & 0 \end{bmatrix} =$$

$$\begin{bmatrix} & E & L & N & A & R \\ E & S_L S_N S_A E r_A & S_N S_A E r_A & S_A E r_A & E r_A & 0 \\ L & S_L \Theta_{LL} C_{LL} h_L + S_L S_N \Theta_{NL} C_{LN} h_N + S_L S_N S_A \Theta_{AL} C_{LA} h_A & S_N \Theta_{NL} C_{LN} h_N + S_N S_A \Theta_{AL} C_{LA} h_A & S_A \Theta_{AL} C_{LA} h_A & 0 & \frac{P_L i N_{LH}}{D_L} \\ N & S_L \Theta_{LN} C_{NL} h_L + S_L S_N \Theta_{NN} C_{NN} h_N + S_L S_N S_A \Theta_{AN} C_{NA} h_A & S_N \Theta_{NN} C_{NN} h_N + S_N S_A \Theta_{AN} C_{NA} h_A & S_A \Theta_{AN} C_{NA} h_A & 0 & \frac{P_N i N_{NH}}{D_N} \\ A & S_L \Theta_{LA} C_{AL} h_L + S_L S_N \Theta_{NA} C_{AN} h_N + S_L S_N S_A \Theta_{AA} C_{AA} h_A & S_N \Theta_{NA} C_{AN} h_N + S_N S_A \Theta_{AA} C_{AA} h_A & S_A \Theta_{AA} C_{AA} h_A & 0 & \frac{P_A i N_{AH}}{D_A} \\ R & S_L Q_L h_L + S_L S_N Q_N h_N + S_L S_N S_A Q_A h_A & S_N Q_N \mathrm{h}_N + S_N S_A Q_A h_A & S_A Q_A h_A & 0 & 0 \end{bmatrix}$$

K11 = The expected number of ticks infected at birth (transovarial), caused by a tick that was infected at birth.

$$K11 = S_L S_N S_A E\ r_A$$

- r_A = Transmission probability from adult to egg.
- E = Number of eggs per female.
- S_L = Survival probability from egg to feeding larva.
- S_N = Survival probability from feeding larva to feeding nymph.
- S_A = Survival probability from feeding nymph to feeding adult.

K12 = The expected number of ticks infected at birth (transovarial), caused by a tick that was infected during a blood meal as a larva.

$$K12 = S_N S_A E\ r_A$$

- r_A = Transmission probability from adult to egg.
- E = Number of eggs per female.
- S_N = Survival probability from feeding larva to feeding nymph.

- S_A = Survival probability from feeding nymph to feeding adult.

K13 = The expected number of ticks infected at birth (transovarial), caused by a tick that was infected during a blood meal as a nymph.

$$K13 = S_A E\, r_A$$

- r_A = Transmission probability from adult to egg.
- E = Number of eggs per female.
- S_A = Survival probability from feeding nymph to feeding adult.

K14 = The expected number of ticks infected at birth (transovarial), caused by a tick that was infected during a blood meal as an adult.

$$K14 = Er_A$$

K15 = The expected number of ticks infected at birth, caused by a viremic host (systemic transmission) = 0.

K21 = The expected number of ticks infected during their first blood meal as larvae, caused by a tick infected at birth (co-feeding).

- S_L = Survival probability from egg to feeding larva.
- S_N = Survival probability from feeding larva to feeding nymph.
- S_A = Survival probability from feeding nymph to feeding adult.
- C_{LL} = Mean no. larvae cofeeding with a larva.
- C_{LN} = Mean no. larvae cofeeding with a nymph.
- C_{LA} = Mean no. larvae cofeeding with an adult.
- h_L = Probability of a larva feeding on a competent host
- h_N = Probability of a nymph feeding on a competent host
- h_A = Probability of an adult feeding on a competent host
- Θ_{LL} = Efficiency from larva to larva.
- Θ_{NL} = Efficiency from nymph to larva.
- Θ_{AL} = Efficiency from adult to larva.

$$K21 = S_L \Theta_{LL} C_{LL} h_L + S_L S_N \Theta_{NL} C_{LN} h_N + S_L S_N S_A \Theta_{AL} C_{LA} h_A$$

K22 = The expected number of ticks infected during their first blood meal as larvae, caused by a tick infected during a blood meal as a larva (co-feeding).

- S_N = Survival probability from feeding larva to feeding nymph.
- S_A = Survival probability from feeding nymph to feeding adult.
- C_{LN} = Mean no. larvae cofeeding with a nymph.
- C_{LA} = Mean no. larvae cofeeding with an adult.
- h_N = Probability of a nymph feeding on a competent host
- h_A = Probability of an adult feeding on a competent host
- Θ_{NL} = Efficiency from nymph to larva.
- Θ_{AL} = Efficiency from adult to larva.

$$K22 = S_N \Theta_{NL} C_{LN} h_N + S_N S_A \Theta_{AL} C_{LA} h_A$$

K23 = The expected number of ticks infected during their first blood meal as larvae, caused by a tick infected during a blood meal as a nymph (co-feeding).

- S_A = Survival probability from feeding nymph to feeding adult.
- C_{LA} = Mean no. larvae cofeeding with an adult.
- h_A = Probability of an adult feeding on a competent host
- Θ_{AL} = Efficiency from adult to larva.

$$K23 = S_A \Theta_{AL} C_{LA} h_A$$

K24 = The expected number of ticks infected during their first blood meal as larvae, caused by a tick infected during a blood meal as an adult (co-feeding) = 0.

K25 = The expected number of ticks infected during their first blood meal as larvae, caused by a viremic host (systemic transmission).

- P_L = Efficiency from competent host to larva.
- i = Systemic infection duration.
- N_{LH} = Average no. larvae on competent hosts.
- D_L = Days of attachment of larva.

$$K25 = \frac{P_L i N_{LH}}{D_L}$$

K31 = The expected number of ticks infected during their second blood meal as nymphs, caused by a tick infected at birth (co-feeding).

- S_L = Survival probability from egg to feeding larva.
- S_N = Survival probability from feeding larva to feeding nymph.
- S_A = Survival probability from feeding nymph to feeding adult.
- C_{NL} = Mean no. nymphs cofeeding with a larva.
- C_{NN} = Mean no. nymphs cofeeding with a nymph.
- C_{NA} = Mean no. nymphs cofeeding with an adult.
- h_L = Probability of a larva feeding on a competent host
- h_N = Probability of a nymph feeding on a competent host
- h_A = Probability of an adult feeding on a competent host
- Θ_{LN} = Efficiency from larva to nymph.
- Θ_{NN} = Efficiency from nymph to nymph.
- Θ_{AN} = Efficiency from adult to nymph.

$$K31 = S_L \Theta_{LN} C_{NL} h_L + S_L S_N \Theta_{NN} C_{NN} h_N + S_L S_N S_A \Theta_{AN} C_{NA} h_A$$

K32 = The expected number of ticks infected during their second blood meal as nymphs, caused by a tick infected during a blood meal as a larva (co-feeding).

- S_N = Survival probability from feeding larva to feeding nymph.
- S_A = Survival probability from feeding nymph to feeding adult.
- C_{NN} = Mean no. nymphs cofeeding with a nymph.
- C_{NA} = Mean no. nymphs cofeeding with an adult.
- h_N = Probability of a nymph feeding on a competent host
- h_A = Probability of an adult feeding on a competent host
- Θ_{NN} = Efficiency from nymph to nymph.
- Θ_{AN} = Efficiency from adult to nymph.

$$K32 = S_N \Theta_{NN} C_{NN} h_N + S_N S_A \Theta_{AN} C_{NA} h_A$$

K33 = The expected number of ticks infected during their second blood meal as nymphs, caused by a tick infected during a blood meal as a nymph (co-feeding).

- S_A = Survival probability from feeding nymph to feeding adult.
- C_{NA} = Mean no. nymphs cofeeding with an adult.
- h_A = Probability of an adult feeding on a competent host
- Θ_{AN} = Efficiency from adult to nymph.

$$K33 = S_A \Theta_{AN} C_{NA} h_A$$

K34 = The expected number of ticks infected during their second blood meal as nymphs, caused by a tick infected during a blood meal as an adult (co-feeding) = 0.

K35 = The expected number of ticks infected during their second blood meal as nymphs, caused by a viremic host (systemic transmission).

- P_N = Efficiency from competent host to nymph.
- i = Systemic infection duration.
- N_{NH} = Average no. nymphs on competent host.
- D_A = Days of attachment of nymph.

$$K35 = \frac{P_N i N_{NH}}{D_N}$$

K41 = The expected number of ticks infected during their third blood meal as adults, caused by a tick infected at birth.

- S_L = Survival probability from egg to feeding larva.
- S_N = Survival probability from feeding larva to feeding nymph.
- S_A = Survival probability from feeding nymph to feeding adult.
- C_{AL} = Mean no. adults cofeeding with a larva.
- C_{AN} = Mean no. adults cofeeding with a nymph.
- C_{AA} = Mean no. adults cofeeding with an adult.
- h_L = Probability of a larva feeding on a competent host
- h_N = Probability of a nymph feeding on a competent host
- h_A = Probability of an adult feeding on a competent host
- Θ_{LA} = Efficiency from larva to adult.
- Θ_{NA} = Efficiency from nymph to adult.
- Θ_{AA} = Efficiency from adult to adult.

$$K41 = S_L \Theta_{LA} C_{AL} h_L + S_L S_N \Theta_{NA} C_{AN} h_N + S_L S_N S_A \Theta_{AA} C_{AA} h_A$$

K42 = The expected number of ticks infected during their third blood meal as adults, caused by a tick infected during a blood meal as a larva (co-feeding).

- S_N = Survival probability from feeding larva to feeding nymph.
- S_A = Survival probability from feeding nymph to feeding adult.
- C_{AN} = Mean no. adults cofeeding with a nymph.
- C_{AA} = Mean no. adults cofeeding with an adult.
- h_N = Probability of a nymph feeding on a competent host
- h_A = Probability of an adult feeding on a competent host
- Θ_{NA} = Efficiency from nymph to adult.
- Θ_{AA} = Efficiency from adult to adult.

$$K42 = S_N \Theta_{NA} C_{AN} h_N + S_N S_A \Theta_{AA} C_{AA} h_A$$

K43 = The expected number of ticks infected during their third blood meal as adults, caused by a tick infected during a blood meal as a nymph (co-feeding).

- S_A = Survival probability from feeding nymph to feeding adult.
- C_{AA} = Mean no. adults cofeeding with an adult.
- h_A = Probability of an adult feeding on a competent host
- Θ_{AA} = Efficiency from adult to adult.

$$K42 = S_A \Theta_{AA} C_{AA} h_A$$

K44 = The expected number of ticks infected during their third blood meal as adults, caused by a tick infected during a blood meal as an adult (co-feeding) = 0.

K45 = The expected number of ticks infected during their third blood meal as adults, caused by a viremic host (systemic transmission) = 0. Roe deer, sheep, goats, cattle and other important mammalian tick hosts and birds have not (yet) been proven to be competent hosts supporting non-viraemic transmission between co-feeding ticks [69, 70].

- P_A = Efficiency from competent host to adult.
- i = Systemic infection duration.
- N_{AH} = Average no. adults on competent host.
- D_A = Days of attachment of adults.

$$K45 = \frac{P_A i N_{AH}}{D_A}$$

K51 = The expected number of infected hosts, caused by a tick infected at birth (systemic transmission).

- S_L = Survival probability from egg to feeding larva.
- S_N = Survival probability from feeding larva to feeding nymph.
- S_A = Survival probability from feeding nymph to feeding adult.
- Q_L = Efficiency from larva to competent host.
- Q_N = Efficiency from nymph to competent host.
- Q_A = Efficiency from adult to competent host.
- h_L = Probability of a larva feeding on a competent host
- h_N = Probability of a nymph feeding on a competent host
- h_A = Probability of an adult feeding on a competent host

$$K51 = S_L Q_L h_L + S_L S_N Q_N h_N + S_L S_N S_A Q_A h_A$$

K52 = The expected number of infected hosts, caused by a tick infected as larvae (systemic transmission).

- S_N = Survival probability from feeding larva to feeding nymph.
- S_A = Survival probability from feeding nymph to feeding adult.
- Q_N = Efficiency from nymph to competent host.
- Q_A = Efficiency from adult to competent host.
- h_N = Probability of a nymph feeding on a competent host
- h_A = Probability of an adult feeding on a competent host

$$K52 = S_N Q_N \mathrm{h}_N + S_N S_A Q_A h_A$$

K53 = The expected number of infected hosts, caused by a tick infected as nymphs (systemic transmission).

- S_A = Survival probability from feeding nymph to feeding adult.
- Q_A = Efficiency from adult to competent host.
- H_A = Probability of an adult feeding on a competent host

$$K53 = S_A Q_A h_A$$

K54 = The expected number of infected hosts, caused by a tick infected as adults (systemic transmission) = 0.

K55 = The expected number of infected hosts, caused by an infected host = 0.

Terms described in Supplementary Table S5 were assumed to be spatially independent, i.e. constant over all grid cells. We based our estimates of the transmission efficiency between ticks and wild rodents on an unpublished dataset referred to in [56] because of a lack of studies. Additionally, with no known differences in viraemia levels and duration between bank voles and mice from the *Apodemus* genus [71], we assumed an equal duration of systemic infection for all four rodent species (i.e., bank voles, short-tailed field voles, yellow-necked field mice, and wood mice). Tick attachment duration and efficiency rates were assumed to be the same for all four rodents [57].

The survival probability from one life stage to the next depends on whether a tick is able to find a host [57]. We adapted the framework provided by [57] to estimate the probability of survival based on the combined density of birds and rodents. We used a simple linear model to predict survival probabilities given a certain density which were not included in the scenarios used in [57] (Supplementary Figure S5). From these trendlines, we interpolated the survival probabilities across our range of bird and rodent densities. To our knowledge, no estimates were available for changes in survival probability from egg to feeding larva; we therefore assumed these probabilities would follow a similar trend as those for survival from feeding larva to feeding nymph (Supplementary Figure S5). Survival probability from feeding nymph to feeding adult was held constant as done in (Supplementary Table S5) [57]. The number of ticks of a given life stage detected on a certain number of hosts was extracted from the database available in [57]. For each life stage (i.e., larva, nymph, and adult), we calculated the weighted mean tick burden per host and the maximum value (Supplementary Table S6). The maximum value was defined as the weighted 99.9th percentile of the distribution of tick counts for a given life stage recorded on a host species. Weights for the weighted mean were based on the number of hosts examined. In case of some species only one or a few papers were available in the database; papers containing too extreme values in tick burden were therefore not included [57]. Papers that did not specify the life stage detected were not included either. We assumed that grid cells with 100% habitat suitability hosted the maximum number of ticks per host and we therefore calculated the tick burden for a host in a grid cell as the product of the proportion of suitable habitat and the maximum tick burden.

Co-feeding between life stages was calculated as a function of the mean number of ticks of life stage k per grid cell i (X_{ki}) and the density of rodent host species j per grid cell i. The number of ticks of life stage k on host species j in grid cell i, was calculated using equation 1. Where D_{ji} denotes the density of host species j in grid cell i and N_{kji} denotes the number of ticks of life stage k on host species j in grid cell i. We then calculated co-feeding between life stage k and t using equation 2, where N_{tji} was the number of ticks of life stage t on host species j in grid cell i. To the best of our knowledge co-feeding has not been demonstrated on larger mammals and birds. As a result, the summation in equations 1 and 2 was limited to the bank vole, short-tailed field vole, yellow-necked field mouse, and wood mouse. Co-feeding between adult life was assumed to be epidemiologically and ecologically irrelevant TBEV, and was thus not considered [56, 72].

$$X_{ki} = \sum_{j=1}^{n=4} (D_{ji}\, N_{kji}) \qquad \text{(Equation 1)}$$

$$C_{kti} = \sum_{j=1}^{n=4} \frac{N_{kji}/D_{ji}}{X_{ki}} \times N_{tji} \qquad \text{(Equation 2)}$$

The fraction of blood meals taken on a competent host depends (h) on the host community composition (e.g., number of birds and mammals) of a grid cell (equation 3). In equation 3, N_{kji} represented the number of ticks of life stage k on host species j in grid cell i, and D_{ji} represented the density of host species j in grid cell i. Set C included only competent hosts (i.e., rodent species), whereas set A included all host types.

$$h_{ki} = \frac{\sum_{j \in C} N_{kji} D_{ji}}{\sum_{j \in A} N_{kji} D_{ji}} \qquad \text{(Equation 3)}$$

Nine scenarios were used to explore how the R_0-value spatially varied based on host community (Supplementary Table S7).

Supplementary Tables S3. Epidemiological modelling and scenarios

Table S5. Spatially independent parameters derived from literature.

Terms	Description	Estimate
r_a	Transmission probability from adult to egg	0.001 [a]
E	Number of eggs per female	2,000 [b]
θ	Efficiency from tick to tick	0.55 [c, d]
D_L	Days of attachment of larva	3.8 days [e]
D_N	Days of attachment of nymph	5.3 days [e]
D_A	Days of attachment of adult	6.9 days [e]
i	Systemic infection duration in rodents	2 days [f, g, h]
p_l	Efficiency from competent host to larva	0.8 [i]
p_n	Efficiency from competent host to nymph	0.8 [i]
p_a	Efficiency from competent host to adult	0.8 [i]
q_l	Efficiency from larva to competent host	0.9 [i]
q_n	Efficiency from nymph to competent host	0.9 [i]
q_a	Efficiency from adult to competent host	0.9 [i]
s_a	Survival probability from feeding nymph to feeding adult	0.1 [b, j]

Subscripts a-i correspond to the following sources: a = [73], b = [74], c = [66], d = [64], e = [75], f = [76], g = [77], h = [78], i = [56], and j = [57].

Table S6. Range (weighted mean (maximum)) tick burden on hosts.

Terms	Description		Reference estimate
N_{LRD}	Average number of larvae on host (max) [a]	Roe deer	1.35 (2.87)
N_{LFD}		Fallow deer	0.45 (0.45)
N_{LRF}		Red fox	0 (0)
N_{LBV}		Bank vole	3.05 (12.84)
N_{LSFV}		Short-tailed field vole	0.7 (16.14)
N_{LYFM}		Yellow-necked field mouse	7.05 (19.0)
N_{LWM}		Wood mouse	5.62 (14.60)
N_{LES}		European stonechat	16 (16)
N_{LCB}		Common blackbird	0.76 (4.28)
N_{LTP}		Tree pipit	0.31 (1.0)
N_{LESK}		Eurasian skylark	0.09 (5.60)
N_{LST}		Song thrush	0.49 (4.0)
N_{LD}		Dunnock	0.15 (1.16)
N_{LEJ}		Eurasian jay	0.23 (2.0)
N_{LCS}		Common starling	0.35 (1.83)
N_{LH}		Hawfinch	0.41 (2.85)
N_{LER}		European robin	0.26 (2.0)
N_{LCC}		Common chaffinch	0.5 (7.92)
N_{NRD}	Average number of nymphs on host (max) [a]	Roe deer	6.03 (15.90)
N_{NFD}		Fallow deer	10.91 (10.91)
N_{NRF}		Red fox	0.03 (0.03)
N_{NBV}		Bank vole	0.12 (0.46)
N_{NSFV}		Short-tailed field vole	0.13 (0.91)
N_{NYFM}		Yellow-necked field mouse	0.2 (0.90)
N_{NWM}		Wood mouse	0.09 (0.66)
N_{NES}		European stonechat	0 (0)
N_{NCB}		Common blackbird	1.45 (8.73)
N_{NTP}		Tree pipit	0.14 (0.89)
N_{NESK}		Eurasian skylark	0.02 (0.02)
N_{NST}		Song thrush	0.51 (3.82)
N_{ND}		Dunnock	0.33 (1.64)
N_{NEJ}		Eurasian jay	1.12 (1.22)
N_{NCS}		Common starling	0.11 (0.92)
N_{NH}		Hawfinch	0.49 (2.0)
N_{NER}		European robin	0.13 (0.81)
N_{NCC}		Common chaffinch	0.09 (0.92)
N_{ARD}	Average number of adults on host (max) [a]	Roe deer	15.46 (42.4)
N_{AFD}		Fallow deer	5.73 (6.15)
N_{ARF}		Red fox	2.28 (8.63)
N_{ABV}		Bank vole	0 (0)

N_{ASFV}	Short-tailed field vole	0 (0)
N_{AYFM}	Yellow-necked field mouse	0 (0)
N_{AWM}	Wood mouse	0 (0)
N_{AES}	European stonechat	0 (0)
N_{ACB}	Common blackbird	0 (0)
N_{ATP}	Tree pipit	0 (0)
N_{AESK}	Eurasian skylark	0 (0)
N_{AST}	Song thrush	0 (0)
N_{AD}	Dunnock	0 (0)
N_{AEJ}	Eurasian jay	0 (0)
N_{ACS}	Common starling	0 (0)
N_{AH}	Hawfinch	0 (0)
N_{AER}	European robin	0 (0)
N_{ACC}	Common chaffinch	0 (0)

Subscript a corresponds to [57].

Table S7. R_0-scenarios

Scenarios	*1*	*2*	*3*	*4*	*5*	*6*	*7*	*8*	*9*
Mean range breeding density birds [a]	X		X	X	X	X	X	X	X
No birds		X							
Low-density roe deer [b]			X						
No fallow deer				X					
No red fox					X				
No bank vole						X			
No short-tailed field vole							X		
No yellow-necked field mouse								X	
No wood mouse									X

[a] Baseline scenario includes all host species and groups.
[b] To assess the impact of low densities of roe deer on the value of R_0, roe deer densities were set to zero, while tick habitat suitability was left unadjusted, e.g., grid cells classified as suitable for ticks remained so even when roe deer were removed from the input data. In reality, the absence of roe deer is associated with a substantial decline in tick densities: [79] reported up to a 99% reduction in *Ixodes ricinus* densities in plots without deer.

Supplementary Figures S3. Epidemiological modelling and scenarios

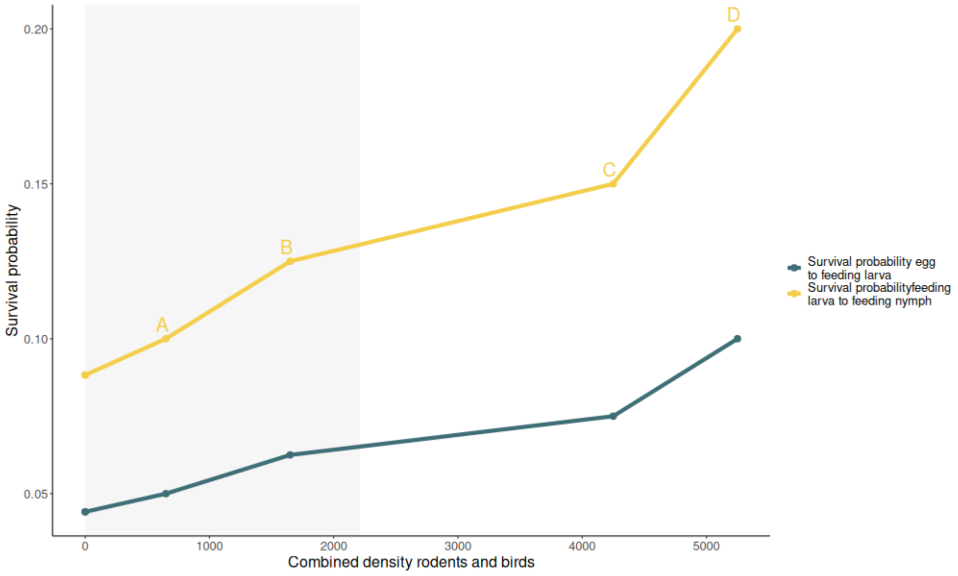

Figure. S5. Survival probability from egg to feeding larva (blue line) and from feeding larva to feeding nymph (yellow line). The letters A (bird, rodent density = 650), B (bird, rodent density = 1,650), C (bird, rodent density = 4,250), D (bird, rodent density = 5,250) indicate the estimates used in [57], the grey area indicates the combined range of rodents and birds in our model. Survival probability from egg to feeding larva (range = 0.04 – 0.10), survival probability from feeding larva to feeding nymph (range = 0.09 – 0.20).

Supplement S4. Model Validation

Supplementary Text S4. Model Validation

The baseline scenario was validated with One Health Monitoring data on the local presence of TBEV in ticks, rodents, and roe deer. These data were extracted from previous literature [80-83]. Alongside these data, an additional effort was made in 2024–25 to assess TBEV seroprevalence in roe deer [84]. Roe deer samples were processed by the Dutch Wildlife Health Centre (DWHC), and analyzed at the National Institute of Public Health and the Environment (RIVM) in the Netherlands following methods described in [81].

Roe deer sera were analyzed using a commercial Enzyme-Linked Immunosorbent Assay (ELISA) (EIA TBEV virus IgG, Testline, Testline Clinical Diagnostics s.r.o. Brno, Czech Republic) according to the manufacturer's instructions. The conjugate was adapted by using a Protein G*HRP conjugate (Thermo Fisher Scientific) for the analysis of roe deer samples. Consequently, the cut-off procedure was also modified. Samples were then characterized as:

1. Negative: if the OD value of the sample was at or below the cut-off value;
2. Positive: if the OD value of the sample greater than or equal to 5 times the SD of the negative controls plus the average OD value for the negative controls, or;
3. Borderline: Samples with OD values above cut-off but below the criteria for positivity.

For model validation, we included only samples in which TBEV was detected (i.e., positive samples from ticks, rodents, or roe deer (positive based on ELISA)). For each of these positive samples, we determined whether the calculated R_0-value in the area where the sample was collected was above or below one. Negative TBEV samples were not included in the validation, because the absence of TBEV in these areas does not necessarily indicate unsuitability (i.e., $R_0<1$) as TBEV simply may not have been introduced yet. In case of rodent and tick samples, we took the calculated R_0-value of the grid cell corresponding to their latitude and longitude coordinates, whereas in case of the roe deer samples we took the maximum R_0-value within a squared buffer area of 76 ha (~ mean home range size at monthly scale (range: 51.4 – 136.0 ha) [85]) around the location where they were shot. This was done to account for the fact that roe deer are not always shot in the area they spend most of their time. We applied a permutation test with 10,000 iterations to test whether the observed proportion of TBEV-positive locations with a $R_0\geq1$ was significantly higher than what would be expected by chance.

Supplementary Tables S4. Model validation

Table S8. One Health Monitoring data (roe deer (*Capreolus capreolus*), small rodent species (*Apodemus* spp., *Clethrionomys glareolus*, *Microtus agrestis)*, and *Ixodes ricinus* ticks) and corresponding basic reproduction values (R_0).

ID sample	Sample type	Classification	R_0
**132**	TBEV in rodents	R0≥1	1.83
**135**	TBEV in rodents	R0≥1	1.83
**182**	TBEV in ticks	R0≥1	1.75
**189**	TBEV seropositivity in roe deer	R0≥1	1.86
**19–3001**	TBEV in rodents	R0<1	0.91
**19–3002**	TBEV in rodents	R0<1	0.91
**19–3053**	TBEV in rodents	R0≥1	1.71
**2**	TBEV in ticks	R0≥1	1.16
**2018-TA226**	TBEV in ticks	R0≥1	1.83
**2019-4040**	TBEV in ticks	R0≥1	1.86
**214**	TBEV seropositivity in roe deer	R0≥1	1.82
**262**	TBEV seropositivity in roe deer	R0≥1	1.85

**285**	TBEV seropositivity in roe deer	R0<1	0.72
**298**	TBEV seropositivity in roe deer	R0<1	0.37
**3**	TBEV in ticks	R0≥1	1.57
**322**	TBEV in rodents	R0<1	0.91
**366**	TBEV seropositivity in roe deer	R0<1	0.91
**379**	TBEV seropositivity in roe deer	R0≥1	1.6
**394**	TBEV in ticks	R0≥1	1.75
**395**	TBEV in ticks	R0≥1	1.75
**399**	TBEV in ticks	R0≥1	1.75
**407**	TBEV seropositivity in roe deer	R0≥1	1.5
**426**	TBEV in ticks	R0≥1	1.75
**428**	TBEV in ticks	R0≥1	1.75
**469**	TBEV in ticks	R0≥1	1.75
**471**	TBEV in ticks	R0≥1	1.75
**472**	TBEV in ticks	R0≥1	1.75
**473**	TBEV in ticks	R0≥1	1.75
**474**	TBEV in ticks	R0≥1	1.75
**475**	TBEV in ticks	R0≥1	1.75
**476**	TBEV in ticks	R0≥1	1.75
**477**	TBEV in ticks	R0≥1	1.75
**478**	TBEV in ticks	R0≥1	1.75
**479**	TBEV in ticks	R0≥1	1.75
**480**	TBEV in ticks	R0≥1	1.75
**486**	TBEV seropositivity in roe deer	R0<1	0.98
**542**	TBEV seropositivity in roe deer	R0<1	0.28
**543**	TBEV seropositivity in roe deer	R0≥1	1.65
**570**	TBEV seropositivity in roe deer	R0<1	0.82
**612**	TBEV seropositivity in roe deer	R0≥1	1.07
**650**	TBEV seropositivity in roe deer	R0≥1	1.01
**661**	TBEV seropositivity in roe deer	R0≥1	1.46
**686**	TBEV seropositivity in roe deer	R0≥1	2.11
**692**	TBEV seropositivity in roe deer	R0≥1	1.82
**70**	TBEV seropositivity in roe deer	R0≥1	1.44
**755**	TBEV seropositivity in roe deer	R0≥1	1.7
**798**	TBEV seropositivity in roe deer	R0<1	0.88
**806**	TBEV seropositivity in roe deer	R0<1	0.56
**817**	TBEV seropositivity in roe deer	R0≥1	1.35
**82**	TBEV in rodents	R0≥1	1.75
**84**	TBEV seropositivity in roe deer	R0≥1	1.97
**898**	TBEV seropositivity in roe deer	R0≥1	2.11
**906**	TBEV seropositivity in roe deer	R0≥1	1.33
**911**	TBEV seropositivity in roe deer	R0≥1	1.13
**912**	TBEV seropositivity in roe deer	R0≥1	1.8
**918**	TBEV seropositivity in roe deer	R0≥1	1.9
**94**	TBEV in ticks	R0≥1	1.75
**95**	TBEV in ticks	R0≥1	1.75

**96**	TBEV in ticks	R0≥1	1.75
**97**	TBEV in ticks	R0≥1	1.75
**Bloemendaal 2023_675**	TBEV in ticks	R0<1	0.91
**DZIF23_597**	TBEV in ticks	R0≥1	1.62
**Dronten_182**	TBEV in ticks	R0≥1	1.75
**Ear_132**	TBEV in ticks	R0≥1	1.83
**Ear_322**	TBEV in ticks	R0≥1	1.1
**LC171402**	TBEV in ticks	R0≥1	1.36
**MH021184**	TBEV in ticks	R0≥1	1.58
**MZ969636**	TBEV in ticks	R0≥1	1.83
**MZ969637**	TBEV in ticks	R0<1	0.88
**MZ969638**	TBEV in ticks	R0≥1	1.75
**MZ969639**	TBEV in ticks	R0≥1	1.05
**NL2018-14**	TBEV in ticks	R0≥1	1.69
**NL_Salland_348**	TBEV in ticks	R0≥1	1.36
**Ree2400002**	TBEV seropositivity in roe deer	R0≥1	1.06
**Ree2400007**	TBEV seropositivity in roe deer	R0≥1	1.06
**Ree2400012**	TBEV seropositivity in roe deer	R0≥1	1.25
**Ree2400015**	TBEV seropositivity in roe deer	R0<1	0.85
**Ree2400045**	TBEV seropositivity in roe deer	R0≥1	1.67
**Ree2400049**	TBEV seropositivity in roe deer	R0≥1	1.78
**Ree2400091**	TBEV seropositivity in roe deer	R0≥1	1.93
**Ree2400096**	TBEV seropositivity in roe deer	R0≥1	1.9
**Ree2400108**	TBEV seropositivity in roe deer	R0≥1	1.93
**Ree2400127**	TBEV seropositivity in roe deer	R0≥1	1.97
**Ree2400133**	TBEV seropositivity in roe deer	R0<1	0.81
**Ree2400146**	TBEV seropositivity in roe deer	R0≥1	1.97
**Ree2400189**	TBEV seropositivity in roe deer	R0≥1	1.51
**Ree2400191**	TBEV seropositivity in roe deer	R0<1	0.34
**Ree2400195**	TBEV seropositivity in roe deer	R0≥1	1.57
**Ree2400202**	TBEV seropositivity in roe deer	R0≥1	1.49
**Ree2400248**	TBEV seropositivity in roe deer	R0≥1	1.68
**Ree2400261**	TBEV seropositivity in roe deer	R0≥1	1.11
**Ree2400262**	TBEV seropositivity in roe deer	R0≥1	1.36
**Ree2400266**	TBEV seropositivity in roe deer	R0≥1	1.23
**Ree2400296**	TBEV seropositivity in roe deer	R0<1	0.88
**Ree2400297**	TBEV seropositivity in roe deer	R0≥1	1.25
**Ree2400307**	TBEV seropositivity in roe deer	R0≥1	1.7
**Ree2400309**	TBEV seropositivity in roe deer	R0≥1	1.62
**Ree2400323**	TBEV seropositivity in roe deer	R0≥1	1.26
**TBEV in rodents_4**	TBEV in rodents	R0≥1	1.75
**TBEV in ticks_4**	TBEV in ticks	R0≥1	1.75

Supplement S5. Targeted Monitoring

Supplementary Text S5. TBEV Expansion Assessment Based on Roe Deer Samples

Following the methods described in [81], we investigated the spatial expansion of TBEV in the Netherlands in relation to the distribution of foci previously reported [81, 83]. For this analysis, we included serum samples from roe deer collected between 2024 and 2025 (N=312). Because sampling intensity can influence the detection rate of foci [81]; we randomly selected a total of 297 samples, as done in [81]. As done in [81], ELISA positive samples were additionally tested using a serum neutralization test (SNT). Samples positive via SNT were used for the construction of foci. Foci were estimated based on the distance between the samples, with samples located within 10 km of each other grouped into the same foci. We used 100,000 iterations to generate a probability distribution for the estimated number of foci.

Supplementary Text S5. Targeted Monitoring in Ticks

In April (week 18) 2025, May (week 19) 2025, and September (week 36) 2025, ticks were collected from ten locations (Zeeland = 1, North Brabant = 4, Gelderland = 1, Drenthe = 3, Groningen = 1) by dragging a white cotton cloth over the vegetation (1 m^2). Locations were selected based on the seroprevalence status of roe deer (2010, 2017, 2024-25) (N=9) or the detection of a human case (N=1) and the calculated R_0-value under the baseline scenario. All ticks attached to the cotton cloth were collected and stored in 0.2mL Polymerase Chain Reaction (PCR) strip tubes (N=). Female and male ticks were placed in individual tubes; nymphs and larvae were pooled in groups of five. Ticks were transported alive to the RIVM and stored at -80°C. The presence of TBEV in ticks was assessed at the RIVM following methods described in [86]. For analysis, ticks were pooled based on life stage and sex: nymphs and larvae were pooled in groups of 25, males in groups of eight, and females in groups of four. Observational data on TBEV detection in ticks has been displayed in Figure S6.

Supplementary Figure S5. Targeted Monitoring in Ticks

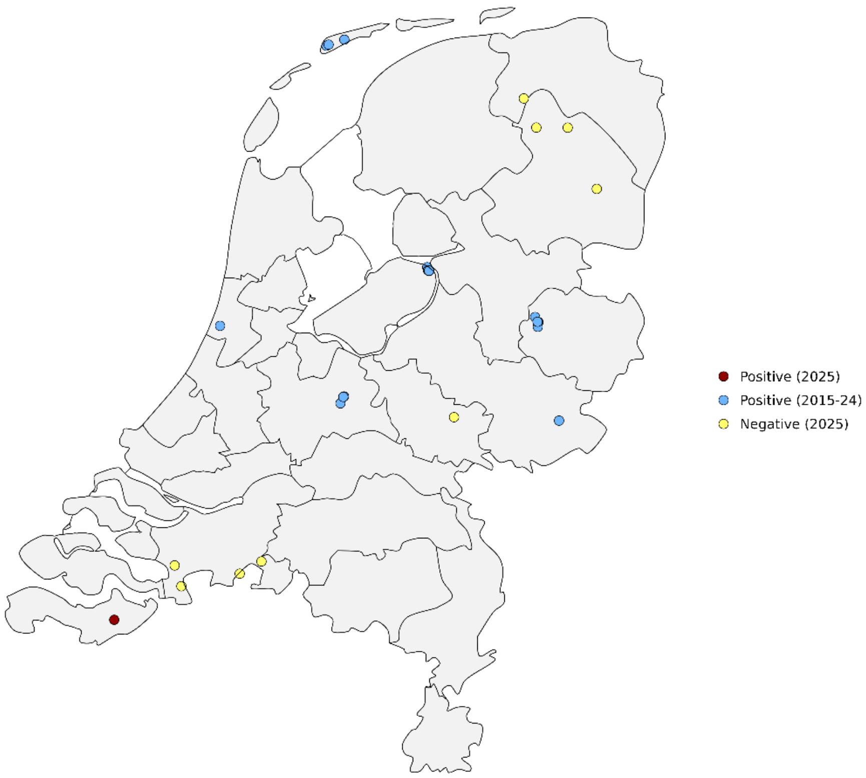

Figure S6. Observational data on TBEV in *Ixodes ricinus* ticks, aggregated by municipal health service (MHS) region in the Netherlands. Data sources include literature-based records from 2015-24, as well as newly collected samples from 2025.

References

1. Jung M, Dahal PR, Butchart SH, Donald PF, De Lamo X, Lesiv M, et al. A global map of terrestrial habitat types. Scientific data. 2020;7(1):256.
2. IUCN. Long-tailed Field Mouse 2023 [Available from: https://www.iucnredlist.org/species/1904/221788084.
3. IUCN. Bank Vole 2023 [Available from: https://www.iucnredlist.org/species/4973/221784180.
4. IUCN. European Roe Deer 2008 [Available from: https://www.iucnredlist.org/species/42395/22161386.
5. IUCN. Common Fallow Deer 2008 [Available from: https://www.iucnredlist.org/species/42188/10656554.
6. IUCN. Red Fox 2006 [Available from: https://www.iucnredlist.org/species/23062/9414323.
7. IUCN. Short-tailed Field Vole 2023 [Available from: https://www.iucnredlist.org/species/221684458/221685769.
8. IUCN. Yellow-necked Field Mouse 2016 [Available from: https://www.iucnredlist.org/species/1892/197269879.
9. GBIF.org. GBIF Occurrence Download - Apodemus flavicollis (Melchior, 1834).
10. GBIF.org. GBIF Occurrence Download - Vulpes vulpes (Linnaeus, 1758).
11. GBIF.org. GBIF Occurrence Download - Capreolus capreolus (Linnaeus, 1758).
12. GBIF.org. GBIF Occurrence Download - Dama dama (Linnaeus, 1758).
13. GBIF.org. GBIF Occurrence Download - Myodes glareolus (Schreber, 1780).
14. GBIF.org. GBIF Occurrence Download - Apodemus sylvaticus (Linnaeus, 1758).
15. GBIF.org. GBIF Occurrence Download - Microtus agrestis (Linnaeus, 1761).
16. Drenthe F, Bruinderink GGWG, Boerema MDL. Faunabeheerplan Ree Drenthe 2024-2029.
17. Overijssel F. Faunabeheerplan Overijssel 2024-2029. Stichting Faunabeheereenheid Overijssel; 2024.
18. Gelderland F. Faunabeheerplan Grote Hoefdieren FBE Gelderland 2019–2025. FBE Gelderland. 2019.
19. van Koot HG, Rosing J, van den Brink B, Jonge Poerink B. Faunabeheerplan Groningen 2019 - 2024. Faunabeheereenheid Groningen; 2019.
20. Schoon CF, Boerema MDL. Faunabeheerplan ree 2020-2024. 2020.
21. Zeeland F. Faunabeheerplan Damhert 2020 – 2025 Zeeland. 2019.
22. Noord-Brabant F. Faunabeheerplan Noord-Brabant 2023-2029. 2023.
23. Zeeland F. Faunabeheerplan Ree 2024-2029. 2023.
24. Zuid-Holland N-e. Faunabeheerplan Damherten.
25. Limburg F. Faunabeheerplan 2020-2026. 2020.
26. Zuid-Holland F. Faunabeheerplan ree Zuid-Holland 2020-2026. 2020.
27. Noord-Holland F. Faunabeheerplan Ree Periode 2021-2026. 2021.
28. Utrecht F. Faunabeheerplan Utrecht 2019-2025. 2019.
29. Flevoland F. Faunabeheerplan Grote hoefdieren 2024-2028. 2023.
30. Noord-Holland F. Rapportage beheer en telling damherten Beheerseizoen 2023-2024. 2024.
31. Zeeland F. Telrapportage 2024. 2024.
32. BureauBiota. De vos [Available from: https://www.bureaubiota.com/informatievoorzieningen/natuurinfotafels/ecoduct-

meedenpad-groningen/paneelvos/#:~:text=Over%20de%20hele%20wereld%20kun,de%20120.000%20en%20140.000%20vossen.
33. Zoogdiervereniging. zoogdiersoorten in Nederland 2024 [Available from: https://www.zoogdiervereniging.nl/zoogdiersoorten.
34. Phillips SJ, Anderson RP, Schapire RE. Maximum entropy modeling of species geographic distributions. Ecological modelling. 2006;190(3-4):231–59.
35. Castillo DSC, Higa M. Strengthening ecologically based rodent management in the Philippines using maximum entropy (MaxEnt) predictions. Journal of Tropical Ecology. 2024;40:e19.
36. Hijmans RJ, Phillips S, Leathwick J, Elith J, Hijmans MRJ. Package 'dismo'. Circles. 2017;9(1):1–68.
37. Fick SE, Hijmans RJ. WorldClim 2: new 1-km spatial resolution climate surfaces for global land areas. International journal of climatology. 2017;37(12):4302–15.
38. Hofmeester T, Coipan E, Van Wieren S, Prins H, Takken W, Sprong H. Few vertebrate species dominate the Borrelia burgdorferi sl life cycle. Environmental Research Letters. 2016;11(4):043001.
39. Sovon. Bird atlas Netherlands: breeding and wintering birds and 40 years of change. Utrecht/Antwerpen: Kosmos Uitgevers; 2018.
40. Sovon. European Stonechat 2018 [Available from: https://stats.sovon.nl/stats/soort/11390.
41. Sovon. Common Blackbird 2018 [Available from: https://stats.sovon.nl/stats/soort/11870.
42. Sovon. Tree pipit 2018 [Available from: https://stats.sovon.nl/stats/soort/10090.
43. Sovon. Eurasian Skylark 2018 [Available from: https://stats.sovon.nl/stats/soort/9760.
44. Sovon. Song Thrush 2018 [Available from: https://stats.sovon.nl/stats/soort/12000.
45. Sovon. Dunnock 2018 [Available from: https://stats.sovon.nl/stats/soort/10840.
46. Sovon. Eurasian Jay 2018 [Available from: https://stats.sovon.nl/stats/soort/15390.
47. Sovon. Common Starling 2018 [Available from: https://stats.sovon.nl/stats/soort/15820.
48. Sovon. Hawfinch 2018 [Available from: https://stats.sovon.nl/stats/soort/17170.
49. Sovon. European Robin 2018 [Available from: https://stats.sovon.nl/stats/soort/10990.
50. Sovon. Common Chaffinch 2018 [Available from: https://stats.sovon.nl/stats/soort/16360.
51. Lindström A, Jaenson TG. Distribution of the common tick, Ixodes ricinus (Acari: Ixodidae), in different vegetation types in southern Sweden. Journal of medical entomology. 2003;40(4):375–8.
52. Uspensky I. Preliminary observations on specific adaptations of exophilic ixodid ticks to forests or open country habitats. Experimental & applied acarology. 2002;28:147–54.
53. Esser HJ, Liefting Y, Ibáñez-Justicia A, Van Der Jeugd H, Van Turnhout CA, Stroo A, et al. Spatial risk analysis for the introduction and circulation of six arboviruses in the Netherlands. Parasites & Vectors. 2020;13:1–20.
54. Swart A, Ibañez-Justicia A, Buijs J, van Wieren SE, Hofmeester TR, Sprong H, et al. Predicting tick presence by environmental risk mapping. Frontiers in public health. 2014;2:238.

55. Hazeu G, Schuiling C, Thomas D, Vittek M, Storm M, Bulens J. Landelijk Grondgebruik Nederland 2021 (LGN2021). 1 ed2023.
56. Hartemink N, Randolph S, Davis S, Heesterbeek J. The basic reproduction number for complex disease systems: Defining R 0 for tick-borne infections. The American Naturalist. 2008;171(6):743–54.
57. Fabri ND, Heesterbeek H, Cromsigt JP, Ecke F, Sprong H, Nijhuis L, et al. Exploring the influence of host community composition on the outbreak potential of Anaplasma phagocytophilum and Borrelia burgdorferi sl. Ticks and Tick-borne Diseases. 2024;15(1):102275.
58. Diekmann O, Heesterbeek JAP. Mathematical epidemiology of infectious diseases: model building, analysis and interpretation: John Wiley & Sons; 2000.
59. Valarcher J, Hägglund S, Juremalm M, Blomqvist G, Renström L, Zohari S, et al. Tick-borne encephalitis. Rev Sci Tech. 2015;34(2):453–66.
60. Matser A, Hartemink N, Heesterbeek H, Galvani A, Davis S. Elasticity analysis in epidemiology: an application to tick-borne infections. Ecology Letters. 2009;12(12):1298–305.
61. Slovák M, Kazimírová M, Siebenstichová M, Ustaníková K, Klempa B, Gritsun T, et al. Survival dynamics of tick-borne encephalitis virus in Ixodes ricinus ticks. Ticks and tick-borne diseases. 2014;5(6):962–9.
62. Danielová V, Holubová J. Transovarial transmission rate of tick-borne encephalitis virus in Ixodes ricinus ticks. Modern acarology. 1991;2:7–10.
63. Rosà R, Tagliapietra V, Manica M, Arnoldi D, Hauffe HC, Rossi C, et al. Changes in host densities and co-feeding pattern efficiently predict tick-borne encephalitis hazard in an endemic focus in northern Italy. International journal for parasitology. 2019;49(10):779–87.
64. Labuda M, Kozuch O, Zuffová E, Elecková E, Hails RS, Nuttall PA. Tick-borne encephalitis virus transmission between ticks cofeeding on specific immune natural rodent hosts. Virology. 1997;235(1):138–43.
65. Labuda M, Austyn JM, Zuffova E, Kozuch O, Fuchsberger N, Lysy J, et al. Importance of localized skin infection in tick-borne encephalitis virus transmission. Virology. 1996;219(2):357–66.
66. Labuda M, Jones LD, Williams T, Danielova V, Nuttall PA. Efficient transmission of tick-borne encephalitis virus between cofeeding ticks. Journal of medical entomology. 1993;30(1):295–9.
67. Daniel M, Danielová V, Fialová A, Malý M, Kříž B, Nuttall PA. Increased relative risk of tick-borne encephalitis in warmer weather. Frontiers in cellular and infection microbiology. 2018;8:90.
68. Süss J. Tick-borne encephalitis 2010: epidemiology, risk areas, and virus strains in Europe and Asia—an overview. Ticks and tick-borne diseases. 2011;2(1):2–15.
69. Nuttall P, Labuda M. Dynamics of infection in tick vectors and at the tick-host interface. Adv Virus Res. 2003;60:233–72.
70. Randolph S, Gern L, Nuttall P. Co-feeding ticks: epidemiological significance for tick-borne pathogen transmission. Parasitology today. 1996;12(12):472–9.
71. Bakker JW, Pascoe EL, van de Water S, van Keulen L, de Vries A, Woudstra LC, et al. Infection of wild-caught wood mice (Apodemus sylvaticus) and yellow-necked mice (A. flavicollis) with tick-borne encephalitis virus. Scientific Reports. 2023;13(1):21627.

72. Randolph S, Miklisova D, Lysy J, Rogers D, Labuda M. Incidence from coincidence: patterns of tick infestations on rodents facilitate transmission of tick-borne encephalitis virus. Parasitology. 1999;118(2):177–86.
73. Danielová V, Holubová J, Pejcoch M, Daniel M. Potential significance of transovarial transmission in the circulation of tick-borne encephalitis virus. Folia parasitologica. 2002;49(4):323–5.
74. Randolph SE, Craine NG. General framework for comparative quantitative studies on transmission of tick-borne diseases using Lyme borreliosis in Europe as an example. Journal of medical entomology. 1995;32(6):765–77.
75. Militzer N, Bartel A, Clausen P-H, Hoffmann-Köhler P, Nijhof AM. Artificial feeding of all consecutive life stages of Ixodes ricinus. Vaccines. 2021;9(4):385.
76. Randolph SE. The shifting landscape of tick-borne zoonoses: tick-borne encephalitis and Lyme borreliosis in Europe. Philosophical Transactions of the Royal Society of London Series B: Biological Sciences. 2001;356(1411):1045–56.
77. Kopecký J, Tomková E, Vlcek M. Immune response of the long-tailed field mouse (Apodemus sylvaticus) to tick-borne encephalitis virus infection. Folia Parasitologica. 1991;38(3):275–82.
78. Kozuch O, Chunikhin S, Gresikova M, Nosek J, Kurenkov V, Lysý J. Experimental characteristics of viraemia caused by two strains of tick-borne encephalitis virus in small rodents. Acta virologica. 1981;25(4):219–24.
79. Hofmeester TR, Sprong H, Jansen PA, Prins HH, Van Wieren SE. Deer presence rather than abundance determines the population density of the sheep tick, Ixodes ricinus, in Dutch forests. Parasites & vectors. 2017;10(1):1–8.
80. Esser HJ, Lim SM, de Vries A, Sprong H, Dekker DJ, Pascoe EL, et al. Continued circulation of tick-borne encephalitis virus variants and detection of novel transmission foci, the Netherlands. Emerging infectious diseases. 2022;28(12):2416.
81. Rijks JM, Montizaan MG, Bakker N, de Vries A, Van Gucht S, Swaan C, et al. Tick-borne encephalitis virus antibodies in roe deer, the Netherlands. Emerging infectious diseases. 2019;25(2):342.
82. Pascoe EL, Bakker JW, Wijburg SR, de Vries A, Sprong H, Marcantonio M, et al. Multiple variants of tick-borne encephalitis virus in voles, mice and ticks, the Netherlands, 2021 to 2023. Eurosurveillance. 2025;30(4):2400247.
83. Jahfari S, De Vries A, Rijks JM, Van Gucht S, Vennema H, Sprong H, et al. Tick-borne encephalitis virus in ticks and roe deer, the Netherlands. Emerging infectious diseases. 2017;23(6):1028.
84. DWHC. Nationale reeënsurveillance 2024 2024 [Available from: https://dwhc.nl/2024/03/nationale-reeensurveillance-2024/.
85. Morellet N, Bonenfant C, Börger L, Ossi F, Cagnacci F, Heurich M, et al. Seasonality, weather and climate affect home range size in roe deer across a wide latitudinal gradient within Europe. Journal of Animal Ecology. 2013;82(6):1326–39.
86. Schwaiger M, Cassinotti P. Development of a quantitative real-time RT-PCR assay with internal control for the laboratory detection of tick borne encephalitis virus (TBEV) RNA. Journal of Clinical Virology. 2003;27(2):136–45.
